# The combined action of Esrrb and Nr5a2 is essential for murine naïve pluripotency

**DOI:** 10.1242/dev.199604

**Published:** 2021-09-10

**Authors:** Nicola Festuccia, Nick Owens, Almira Chervova, Agnès Dubois, Pablo Navarro

**Affiliations:** 1 Regulatory Dynamics and Cell Identity, MRC London Institute of Medical Sciences (LMS), Faculty of Medicine, Imperial College London, Du Cane Road, London W12 0NN, UK; 2 Epigenomics, Proliferation, and the Identity of Cells, Department of Developmental and Stem Cell Biology, Institut Pasteur, CNRS UMR3738, 75015 Paris, France; 3 Institute of Biomedical and Clinical Science, University of Exeter Medical School, Exeter EX2 5DW, UK

**Keywords:** Pluripotency, Embryonic stem cells, Orphan nuclear receptors, Esrrb, Nr5a2

## Abstract

The maintenance of pluripotency in mouse embryonic stem cells (ESCs) is governed by the action of an interconnected network of transcription factors. Among them, only Oct4 and Sox2 have been shown to be strictly required for the self-renewal of ESCs and pluripotency, particularly in culture conditions in which differentiation cues are chemically inhibited. Here, we report that the conjunct activity of two orphan nuclear receptors, Esrrb and Nr5a2, parallels the importance of that of Oct4 and Sox2 in naïve mouse ESCs. By occupying a large common set of regulatory elements, these two factors control the binding of Oct4, Sox2 and Nanog to DNA. Consequently, in their absence the pluripotency network collapses and the transcriptome is substantially deregulated, leading to the differentiation of ESCs. Altogether, this work identifies orphan nuclear receptors, previously thought to be performing supportive functions, as a set of core regulators of naïve pluripotency.

## Abstract (precis)

**Summary:** Esrrb and Nr5a2, two orphan nuclear receptors, are identified as essential regulators of pluripotency in mouse embryonic stem cells.

## INTRODUCTION

The uncommitted identity of mouse embryonic stem cells (ESCs) is maintained by the activity of a gene regulatory network that strictly depends on the function of Oct4 (Pou5f1) and Sox2 (Yeo and Ng, 2013). Binding DNA in complex with Sox2 (Tapia et al., 2015), Oct4 enables the recruitment of other transcription factors (TFs) at regulatory regions (King and Klose, 2017). In accordance with this, altering the expression of either TF results in the differentiation of ESCs (Masui et al., 2007; Niwa et al., 2000). In line with their role in ESCs, Oct4 and Sox2 are essential for epiblast specification (Avilion et al., 2003; Nichols et al., 1998). Alongside these TFs, a number of auxiliary factors stabilise the pluripotency network. Although their individual depletion often affects the efficiency of self-renewal (Chambers et al., 2007) and modulates Oct4 and Sox2 binding at a subset of regulatory elements (Heurtier et al., 2019), the loss of these TFs does not result in overt differentiation. For these reasons, it is generally considered that the pluripotency network is structured along two distinct modules: the core network, centred on Oct4 and Sox2, and a cohort of supportive TFs.

Among all auxiliary factors, Esrrb is prominent (Festuccia et al., 2018b), because it controls multiple aspects of the molecular wiring of ESC identity. Esrrb is a pivotal mediator of pro-self-renewing signalling cues, operating downstream of the canonical WNT pathway (Martello et al., 2012), and bypassing the dependence of ESCs on the cytokine LIF (Festuccia et al., 2012). This TF also acts as a major gatekeeper of pluripotency, both during early differentiation (Festuccia et al., 2018a) and, conversely, during reprogramming to induced pluripotency (Adachi et al., 2018). In addition, Esrrb orchestrates the recruitment of the transcriptional machinery (Bell et al., 2020; Percharde et al., 2012) and other TFs (Adachi et al., 2018) to key regulatory elements (Whyte et al., 2013). Finally, it maintains sequence-specific DNA binding during mitosis, directly contributing to the stability of pluripotency during cell division (Festuccia et al., 2016), as well as after DNA replication (Owens et al., 2019). Given these characteristics, it is not surprising that the genetic ablation of Esrrb compromises ESCs self-renewal in conventional culture conditions (Festuccia et al., 2012). In contrast, the loss of Esrrb, although detrimental (Adachi et al., 2018; Atlasi et al., 2019), is tolerated in culture conditions that more stringently enforce the maintenance of the undifferentiated state (Martello et al., 2012) by blocking the differentiation cues imparted by ERK signalling and reinforcing the activation of WNT by GSK3b inhibition (Ying et al., 2008). How ESCs accommodate the concomitant disruption of the multiple functions of Esrrb action remains unclear, representing a major gap in our knowledge of the molecular control of pluripotency.

One simple hypothesis to explain how in 2i/LIF ESCs can bypass the requirements for Esrrb is that other TFs may perform a compensatory role. Of note, Esrrb is part of a broad family of TFs, orphan nuclear receptors, that show high sequence and structural homology, and have overlapping developmental functions (Festuccia et al., 2018b). Among these, Nr5a2 binds to nearly identical half-palindromic sequences through a related DNA-binding domain (DBD) presenting a common extension to the conventional zinc fingers of nuclear receptors (Heng et al., 2010; Solomon et al., 2005). Furthermore, Nr5a2 is expressed in ESCs, where it contributes to supporting, or instating, pluripotency (Fujii et al., 2015; Gu et al., 2005; Guo and Smith, 2010; Heng et al., 2010). Hence, we sought to test whether Nr5a2 mitigates the consequences of loss of Esrrb function by concomitantly ablating both TFs in ESCs. Here, we report that the loss of both Esrrb and Nr5a2 leads to the complete abrogation of self-renewal and the induction of differentiation, even in 2i/LIF. These effects are mediated by a collapse of the pluripotency network: Oct4, Sox2 and Nanog binding is acutely lost at most enhancers and ESC-specific gene expression is shut down. Therefore, our results identify Esrrb and Nr5a2 as a set of mutually redundant but essential pluripotency TFs, which form a single regulatory module that parallels in importance Oct4 and Sox2 in mouse ESCs.

## RESULTS

### Esrrb and Nr5a2 are co-expressed in individual ESCs and bind to an overlapping set of regulatory elements

**Fig. 1.**
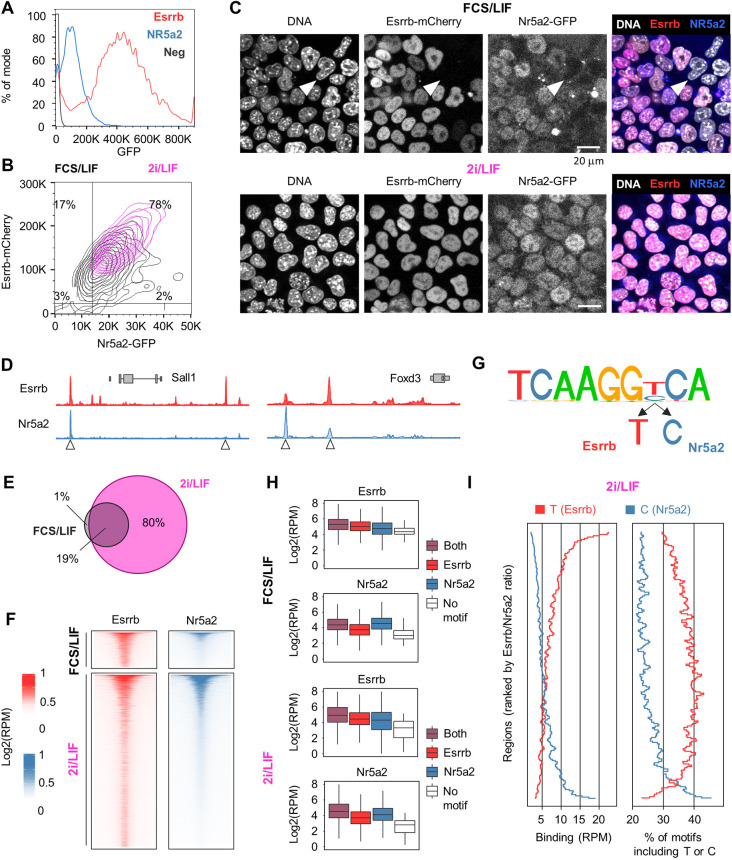
**Overlapping expression and binding pattern of Esrrb and Nr5a2.** (A) Esrrb-T2a-GFP (red) and Nr5a2-T2a-GFP (blue) fluorescence levels (percentage of mode in each dataset) determined by flow cytometry in ESCs cultured in FCS/LIF. The black line shows background levels measured in wild-type E14Tg2a ESCs. Representative of two independent experiments. (B) Nr5a2-GFP and Esrrb-mCherry levels in double knock-in ESCs cultured in FCS/LIF (black) or 2i/LIF (magenta), as determined by imaging flow cytometry (ImageStream). Negative thresholds, identified by analysing wild-type ESCs, are shown (black lines), along with the percentage of cells falling in each gate in FCS/LIF. Results of two pooled independent experiments. (C) Confocal microscopy images showing Esrrb-mCherry and Nr5a2-GFP expression in double knock-in ESCs cultured in FCS/LIF (top) or 2i/LIF (bottom). Note that double-negative cells (white arrowheads) exist only in FCS/LIF. Representative of two independent experiments. (D) Profiles of Esrrb and Nr5a2 binding in FCS/LIF as determined by ChIP-seq in FLAG-Nr5a2 ESCs. The *Sall1* and *Foxd3* loci provide examples of the binding preference of each TF at common targets (arrowheads). (E) Venn diagram showing the overlap between regions bound by Esrrb or Nr5a2 in FCS/LIF and 2i/LIF. (F) Local enrichment heatmap comparing Esrrb (left, red) and Nr5a2 (right, blue) occupancy at regions bound by either of the two TFs in FCS/LIF (top) or 2i/LIF (bottom). Esrrb/Nr5a2 peaks were ordered by decreasing Nr5a2 binding. (G) DNA sequence identified by *de novo* motif discovery at all regions bound by Esrrb/Nr5a2 in FCS/LIF; note the seventh base can either be a T or a C. (H) Box plot showing Esrrb and Nr5a2 binding (RPM, reads per ten million) in FCS/LIF (top) or 2i/LIF (bottom) at target regions containing tcaaggTca, tcaaggCca, both motifs, or none. The central lines correspond to the median, boxes span from the first to the third quartiles, and whiskers extend to the furthest data point within 1.5xIQR from the boxes. The plot highlights how the seventh base of the motif discriminates Esrrb ‘T’ or Nr5a2 ‘C’ preferential binding. (I) Frequency of motifs including T or C at the seventh position (right) at target regions in 2i/LIF, ordered by decreasing Esrrb/Nr5a2 binding ratio (left; RPM, reads per million).

Expression of auxiliary TFs, including Esrrb, is heterogeneous in ESCs cultured in serum and LIF (FCS/LIF), which induces a metastable state permissive for spontaneous differentiation (Chambers et al., 2007). Notably, the loss of Esrrb in this context marks the commitment to the dismantling of pluripotency, triggering the reorganisation of Oct4 binding (Festuccia et al., 2018a). Therefore, we first compared the levels of expression of Esrrb and Nr5a2 in this context. Using GFP inserted in-frame of Nr5a2 or Esrrb, linked by a self-cleaving T2a peptide (Fig. S1), we observed that Esrrb presents a broad distribution of expression levels in ESCs (Fig. 1A), as previously reported. Nr5a2 expression was approximately fivefold weaker, in good agreement with gene expression analysis (Fig. S2A). Importantly, both genes were undetectable in a fraction of the ESC population. Next, we derived additional reporter lines in which the coding sequence for GFP and mCherry have been knocked-in after the Nr5a2 and Esrrb open reading frames (Fig. S1), and found that Esrrb and Nr5a2 proteins show broadly overlapping patterns of expression, with cells negative for one TF also low or negative for the second (Fig. 1B,C). Nonetheless, possibly as a consequence of lower expression, Nr5a2 downregulation was more frequently observed (20% and 5% of Nr5a2 and Esrrb negative cells, respectively), as confirmed by single-cell gene expression analysis (Fig. 1B,C, Fig. S2B,C). In 2i/LIF culture conditions, spontaneous differentiation was suppressed and the expression of Esrrb and other auxiliary TFs reinforced, becoming homogeneous. In line with this, double reporters revealed how in chemically defined medium Esrrb and Nr5a2 protein levels are elevated and their downregulation impeded (Fig. 1B,C, Fig. S2B,C). These results suggest that Esrrb and Nr5a2 respond similarly to signalling cues in ESCs. As the downregulation of Esrrb marks the commitment of ESCs to differentiate, it is possible that Nr5a2 is also relevant in this context, and that the concomitant downregulation of both TFs plays a causal role in the extinction of pluripotency.

To get an indication of whether Esrrb and Nr5a2 play overlapping functions in supporting pluripotency, we established their binding profiles across the genome, in FCS/LIF or 2i/LIF (Table S1), revealing an extensive binding overlap in both conditions (Fig. 1D-F, Fig. S2D). Nr5a2 bound fewer loci in mouse ESCs (FCS/LIF: Esrrb 15556, Nr5a2 6340; 2i/LIF: Esrrb 62786, Nr5a2 36181), possibly owing to its lower expression, particularly in FCS/LIF, and was almost invariably found in association with Esrrb (Fig. 1F). Yet, at common targets, preference for binding of one or the other factor was observed (Fig. 1D, arrowheads). *De novo* motif discovery at regions bound by Esrrb or Nr5a2 found the canonical Esrrb binding consensus – TCAAGGTCA (Festuccia et al., 2016) – with the difference that either T or C could be accommodated at the seventh base of the motif (Fig. 1G). To understand whether variation at this position could explain the preferential recruitment of Esrrb or Nr5a2, we analysed binding associated with each version of the consensus (TCAAGG T/C CA), revealing a preference of Esrrb for T and, more pronouncedly, of Nr5a2 for C (Fig. 1H). This was confirmed by ranking the regions targeted by Esrrb and Nr5a2 by the level of binding of the two factors, which revealed an elevated frequency of motifs including C at the seventh positions at regions with preferential Nr5a2 binding (Fig. 1I, Fig. S2E), and vice versa. Interestingly, whereas perfect matches to the Nr5a2 motif variant accumulated at regions of high Nr5a2, optimal Esrrb motifs were more broadly distributed, and mildly enriched at regions bound by both TFs (Fig. S2F).

Altogether, we conclude that Esrrb and Nr5a2 display similar expression patterns in ESCs, where they bind at a common set of regulatory elements by virtue of highly similar, although specific, DNA-binding preferences.

### Esrrb and Nr5a2 are essential regulators of pluripotency

**Fig. 2.**
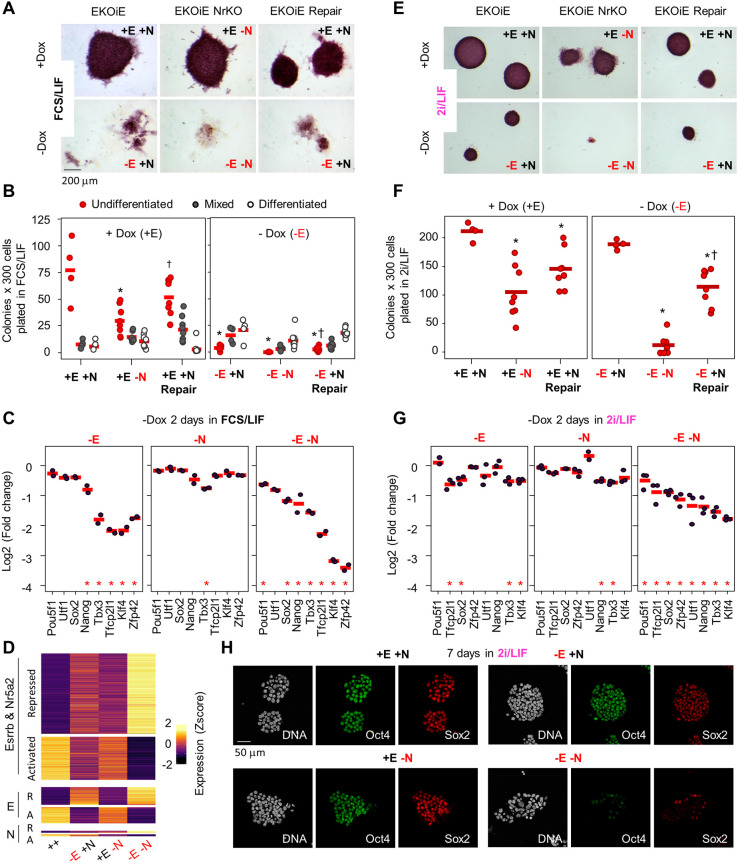
**Esrrb and Nr5a2 are essential for the self-renewal of ESCs and pluripotency gene expression.** (A) Alkaline phosphatase staining of colonies generated by the indicated ESC lines grown in FCS/LIF for 7 days after plating at clonal density. −E+N: loss of Esrrb; +E−N loss of Nr5a2; −E−N loss of both TFs. Representative of four independent experiments. (B) Quantification of the number of undifferentiated, mixed and differentiated colonies in the conditions described in A. Each circle represents an independent experiment (EKOiE *n*=4; other conditions *n*=8); the mean is marked by a red horizontal bar. Asterisks indicate P≤0.05 (Mann–Whitney) for the comparison of each condition to +E+N. Daggers indicate P≤0.05 (Mann–Whitney) for the comparison of +E−N to +E+N Repair or of −E−N to −E+N Repair. (C) RNA-seq fold change of pluripotency gene expression in EKOiE ESCs 2 days after withdrawal of doxycycline (−E; left), in EKOiE NrKO ESCs compared with EKOiE cells (−N; middle), and in EKOiE NrKO ESCs 2 days after withdrawal of doxycycline compared with EKOiE cells (−E−N; right). All cells were grown in FCS/LIF; each circle represents an independent experiment (*n*=2); the mean is marked by a red horizontal line. *FDR<0.01. (D) Heatmap showing the Z-score of transcripts per million (TPM) for genes identified as differentially expressed (absolute fold change >1.5; FDR<0.01) in the conditions indicated on the left; −E, −N and −E−N as in C. Cells were cultured in FCS/LIF. (E-G) Identical analyses to those described in A-C for cells cultured in 2i/LIF (in G, *n*=3). (H) Immunofluorescence showing Oct4 and Sox2 expression in EKOiE (top) and EKOiE NrKO (bottom) ESCs cultured in 2i/LIF in the presence (left) or absence (right) of doxycycline for 7 days. Note the virtually full depletion of Oct4 and Sox2 in the absence of both Esrrb and Nr5a2. Representative of two independent experiments.

We have previously derived ESC lines in which endogenous Esrrb is knocked out and Esrrb expression is rescued by a doxycycline (Dox) inducible transgene (EKOiE), such that upon Dox withdrawal the cells differentiate in FCS/LIF (Festuccia et al., 2016). In these cells, we further disrupted the exon encoding the DBD of Nr5a2 at both alleles, to generate Nr5a2 null ESCs (EKOiE NrKO) (Figs S1, S3A; supplementary Materials and Methods). EKOiE NrKO cells could be derived without special complications, indicating that in this context Nr5a2 is not strictly required for self-renewal (Fujii et al., 2015; Gu et al., 2005; Sladitschek and Neveu, 2019). Indeed, colony-forming assays confirmed that EKOiE NrKO cells self-renew despite showing increased spontaneous differentiation (Fig. 2A,B). In comparison, the loss of Esrrb triggered by Dox withdrawal had more profound effects, resulting in the formation of few undifferentiated colonies, in line with previous results (Festuccia et al., 2012). Notably, the deletion of Nr5a2 exacerbated the effects of Esrrb loss of function, effectively ablating the formation of colonies containing undifferentiated cells 7 days after plating (Fig. 2A,B). This suggests that in FCS/LIF Esrrb plays a preponderant role that is nevertheless supported by Nr5a2. Accordingly, the expression of pluripotency markers was more severely and consistently compromised 2 days after loss of both TFs, than after loss of Esrrb alone (Fig. 2C). These trends were confirmed at the genome-wide level: whereas Esrrb depletion led to 707 differentially expressed genes, the concomitant loss of Nr5a2, which by itself deregulates 91 genes, was accompanied by extensive gene expression changes, with 1666 up- and 903 downregulated genes (FDR<0.01; Fig. 2D, Fig. S3B, Table S2). Moreover, we observed that genes responding to the loss of one orphan receptor, although not passing statistical tests of significance in response to loss of the other, still display concordant expression changes (Fig. 2D, Fig. S3C). As genes activated and repressed by both factors were enriched in terms such as ‘response to LIF’ (FDR=2.27e-24) and in terms linked to differentiation (e.g. ‘morphogenesis’; FDR=2.32e-57; Table S3), respectively, we conclude that Esrrb and Nr5a2 cooperate to support pluripotency in FCS/LIF.

The extensive binding overlap between Esrrb and Nr5a2, their additive effect on gene expression, and the severe phenotype of their deletion in FCS/LIF prompted us to explore the effect of the concomitant loss of function in 2i/LIF, where the invalidation of Esrrb is compatible with self-renewal. In agreement with previous reports (Adachi et al., 2018; Atlasi et al., 2019), clonal plating of EKOiE cells evidenced the detrimental effects of suppressing Esrrb expression, while confirming a non-essential function (Martello et al., 2012) (Fig. 2E,F). In line with this, Esrrb depletion in 2i/LIF resulted in reduced expression of both auxiliary and core pluripotency genes – in particular *Klf4*, *Tbx3*, *Tfcp2l1* and *Sox2* – and in the mild upregulation of differentiation markers (Fig. 2G, Fig. S3D). Yet, expression of Oct4 was unaffected, and colonies of undifferentiated ESCs readily formed. Similarly, the loss of Nr5a2 had detrimental effects, reducing expression of pluripotency genes, the clonogenicity of ESCs and triggering spontaneous differentiation. Nonetheless, it remained tolerated overall, as previously reported (Atlasi et al., 2019). In striking contrast, the concomitant depletion of both TFs completely abolished the capacity of ESCs to self-renew, paralleling the observations made in FCS/LIF. The few remaining colonies showed overt signs of morphological deterioration and included mostly differentiated cells. These effects were specific, as repair of one of the two disrupted Nr5a2 alleles rescued self-renewal (Fig. 2E,F). In accordance with this, gene expression analysis revealed the collapse of pluripotency genes and the upregulation of a panel of differentiation markers just 2 days after acute loss of Esrrb and Nr5a2, an effect that was also reversed after repair (Fig. 2G, Fig. S3D,E). This drastic phenotype was not exclusively observed after clonal plating. In the absence of Esrrb and Nr5a2, ESCs cultures rapidly deteriorated during regular passaging: evident morphological changes preceded a reduction in cell numbers, appearing after day 2 (Fig. S4A-C). In this context, defects in proliferation manifested only once the expression of pluripotency markers had dropped. Therefore, such effects can likely be attributed to differentiation. Immunofluorescence 7 days after Dox withdrawal, also confirmed the near-complete loss of Oct4, Sox2, Nanog and Klf4 in EKOiE NrKO ESCs (Fig. 2H, Fig. S4D). These results reveal a phenotype that is not predictable from the study of single knockouts because it is masked by the functional redundancy between the two orphan receptors. Altogether, we identify Esrrb and Nr5a2 as two arms of a single regulatory unit that is essential for maintenance of pluripotency.

### Loss of Esrrb and Nr5a2 triggers the collapse of the pluripotency network

**Fig. 3.**
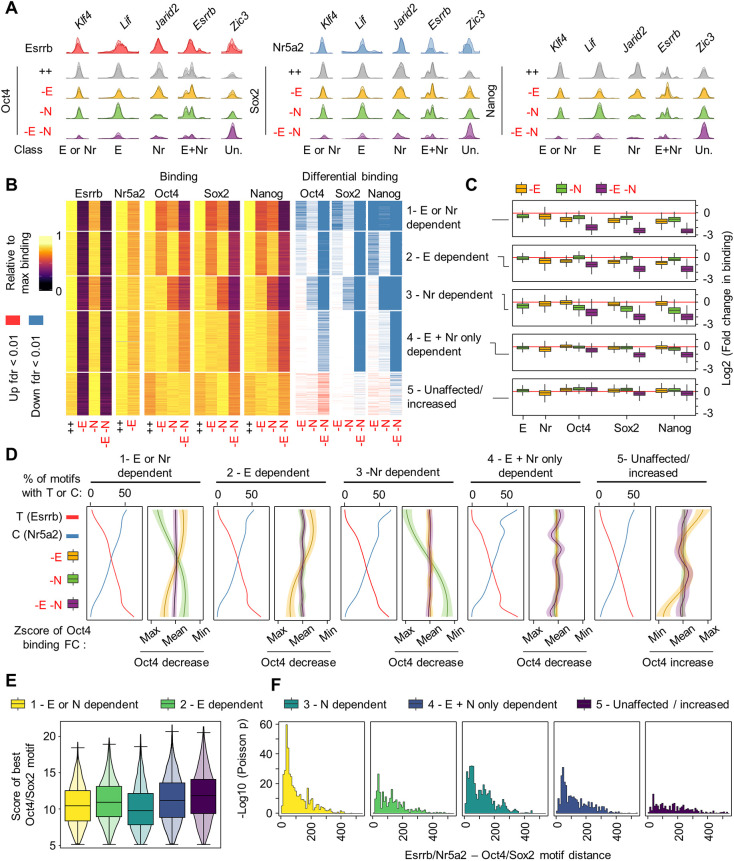
**Esrrb and Nr5a2 control binding of Oct4, Sox2 and Nanog at thousands of regulatory elements.** (A) ChIP-seq profiles of Esrrb, Nr5a2, Oct4, Sox2 and Nanog binding at enhancers in proximity of the indicated genes in EKOiE and EKOiE NrKO ESCs cultured in 2i/LIF with or without doxycycline for 2 days, showcasing elements differentially affected by the depletion of Esrrb (−E), Nr5a2 (−N), or both genes (−E−N). Only for Nr5a2 ChIP, an HA peptide was knocked-in in front of Nr5a2 in EKOiE ESCs. (B) Left: Heatmap showing normalised TF binding levels at regions called as bound by Oct4, Sox2 and Nanog conjunctly with Esrrb or Nr5a2 (for each TF, the condition displaying maximal binding is set to one) in EKOiE (HA-Nr5a2) or EKOiE NrKO ESCs cultured in the presence (++, −N) or absence of doxycycline (−E, −E−N, respectively). The five classes of regions were identified by K-means clustering of Oct4, Sox2 and Nanog occupancy. Right: Calls for significantly increased (red) or decreased (blue) binding of each TF at the same set of regions (FDR<0.05). (C) Box plot, presented as in Fig 1H, showing the fold change in TF binding at the classes of regulatory regions identified in Fig 3B, 2 days after depletion of Esrrb (−E), in the absence of Nr5a2 (−N), and after depleting Esrrb in the absence of Nr5a2 (−E−N). (D) Z-score of the fold change in Oct4 binding at different classes of regulatory regions, ordered the frequency of Esrrb/Nr5a2 motifs containing C versus T at the seventh position, in EKOiE ESCs and EKOiE NrKO ESCs 2 days after depletion of Esrrb and/or Nr5a2 (−E,−N,−E−N as in C). Shaded regions show ±1 standard deviation of the mean of the Gaussian process function. (E) Box plot, overlaid on a violin plot, showing the quality of the best Oct/Sox2 motifs at the different classes of regions identified in B. The box plot is presented as in Fig. 1H. The violin plot shaded areas shows the kernel density estimate of the data. (F) Statistical enrichment (−log10 Poisson *P*-value) of Oct4 motifs in function of the distance to Esrrb/Nr5a2 motifs at the regions identified in B. All pairs of motifs of quality higher than the median were considered.

The profound consequence of the loss of Esrrb and Nr5a2 prompted us to investigate in more detail how these TF conjunctly control pluripotency TF binding, as represented by Oct4, Sox2 and Nanog, in ESCs cultured in 2i/LIF (Table S4). In EKOiE cells grown in the presence of Dox, 77,840 regions were bound by either Esrrb or Nr5a2 (Fig. 3A, Fig. S5A). As expected, Esrrb binding was ablated 2 days after withdrawal of the drug (Fig. 3B). Interestingly, although the loss of Esrrb had a moderate impact on Nr5a2 binding, and the reciprocal effect was even milder (Fig. 3B,C, Fig. S5B), we observed that, in the absence of support by Esrrb, Nr5a2 occupancy levels correlate more directly to the presence of a strong cognate motif at target regions (Fig. S5C). Hence, even though Esrrb and Nr5a2 can bind DNA independently, Esrrb facilitates Nr5a2 occupancy at sites of low affinity, indicating cooperativity in binding.

Next, we assessed the overlap with other pluripotency TFs, and found that more than half of the regions bound conjunctly by Oct4, Sox2 and Nanog were also bound by Esrrb or Nr5a2, in line with the notion that the pluripotency gene regulatory network is extensively interconnected (Fig. S5A). Moreover, although Esrrb and Nr5a2 also bind regions not targeted by the ensemble of other TFs (Festuccia et al., 2016), maximal Esrrb, Nr5a2, Oct4, Sox2 and Nanog binding was observed at shared targets, suggesting a strong global cooperativity (Fig. S5D). Strikingly, we observed that, at these common regulatory nodes, the loss of Esrrb and Nr5a2 leads to a global reduction of TF occupancy (Fig. 3A-C, Fig. S5D,E). Although the reduced levels of Nanog and Sox2 protein 2 days after depletion may partially contribute to these effects, Oct4 remained robustly expressed (Fig. S5F). Moreover, the reduction of Oct4, Sox2 and Nanog binding was significantly stronger at Esrrb/Nr5a2-bound regions than at the ensemble of sites irrespective of Esrrb/Nr5a2 occupancy (Fig. S5D,E). Overall, 43%, 67% and 74% of co-bound regions displayed reduced Oct4, Sox2 and Nanog, respectively, after depletion of Esrrb and Nr5a2. These effects are substantially more severe than those observed after depletion of Nanog in FCS/LIF (Heurtier et al., 2019), and parallel in magnitude the consequence of the loss of Oct4, which reduces accessibility at 72% of its target enhancers (King and Klose, 2017). Importantly, the effects of inactivating Esrrb or Nr5a2 are particularly related. The regions affected by loss of either gene overlapped more extensively – in terms of absolute numbers and enrichment over the intersection expected by chance – than each set did to the group of elements responding to the depletion of Nanog (Fig. S5G,H). We conclude that Esrrb and Nr5a2 are conjunctly required to foster binding of Oct4, Sox2 and Nanog across thousands of shared target regions.

We then sought to dissect further the individual contributions of Esrrb and Nr5a2 to these effects, and identify affected and unaffected regulatory elements. Five major classes of regions were identified (Fig. 3B,C). In the first group, we observed a significant loss of pluripotency TF binding upon the loss of either Esrrb or Nr5a2, and increased effects upon the dual loss. In the second and third groups, all pluripotency factors lost binding in response to the loss of either Esrrb (group 2) or Nr5a2 (group 3), and showed a more pronounced response after their combined depletion. In the fourth and largest group, the single depletions of Esrrb or Nr5a2 were relatively inconsequential and a significant reduction in binding was observed only with the double depletion. Finally, in the fifth group, we found regions that show unaltered or increased levels of Oct4 and Sox2 upon loss of both Esrrb and Nr5a2. Motivated by previous results, we wondered whether these differential dependencies might be explained by the binding preference of the two orphan receptors. Indeed, when we ranked the regions according to the proportion of T/C motifs, we observed a qualitative correlation between this ratio and the magnitude of the effect of Esrrb or Nr5a2 depletion: at regions with more T than C motifs, the effect of Esrrb on Oct4 binding was more pronounced than average, and vice versa. Importantly, at regions with balanced motif prevalence the depletion of either factor led to similar, and average, consequences, as highlighted by the nearly perfectly aligned crossing points of the curves in Fig. 3D. Moreover, such correlations display specificity: for instance, a link between the presence of Nr5a2 motif variants and a reduction in Oct4 was observed only at regions classified as dependent on Nr5a2 alone (group 1 and 3), and exclusively in response to the depletion of this TF.

The fact that Oct4, Sox2 and Nanog occupancy relies on Esrrb and Nr5a2 at some regions, but not others, might also depend on the presence of strong or degenerate binding sites for these TFs, and their distance to those for the two orphan receptors. Indeed, at regions showing dependent TF binding (group 1-4), Oct4/Sox2 composite motifs were more degenerate and more markedly enriched in proximity of Esrrb/Nr5a2 (<100 bp), compared with independent regions (group 5) (Fig. 3E,F). Thus, at these loci, recruitment of Oct4 to weak binding sites might depend on the ability of the two orphan receptors to instate accessibility. Overall, the differential dependencies we observed appear to be linked to the ability of regulatory regions to recruit Esrrb and Nr5a2, and are likely mediated by local effects on the chromatin.

Our findings show that Esrrb and Nr5a2 operate conjunctly at target regulatory elements. Yet the two orphan receptors – as observed for other pluripotency TFs (Dunn et al., 2014) – might simply exert a generically concordant activity. Thus, we set out to identify signs of direct cooperativity between the two TFs. To this end, we identified 163 regions that harbour only one recognisable Esrrb/Nr5a2-binding site. First, we noticed that Esrrb and Nr5a2 reciprocally facilitate each other's access to these shared sites, thus confirming cooperativity in binding (Fig. S5I). Second, we observed that acting at the very same positions on DNA, Esrrb and Nr5a2 affect occupancy by other pluripotency TFs in a greater-than-additive manner (Fig. S5J). These results provide evidence of a direct functional cooperativity, which is distinct from the broad synergy between unrelated TFs.

We further explored the functional significance of the ability of Esrrb and Nr5a2 to affect specific regions. Loci where Esrrb and Nr5a2 facilitate TF binding (clusters 1 to 4) tend to be located close to known pluripotency regulatory elements, such as the enhancers of Klf4, Nanog, Esrrb, Sox2 and Oct4. Globally, these regions were enriched in proximity of genes associated with pluripotency, such as ‘response to LIF signalling’ (FDR<7.17e-7; all four clusters), ‘blastocyst formation’ (FDR=3.8e-4, 3.4e-12; clusters 2 and 4) or ‘stem cell population maintenance’ (FDR=6.2e-9, 1.9e-16; clusters 3 and 4). In contrast, cluster 5 was associated with genes linked to early epiblast differentiation, such as *Grhl3*, *Zic2/3/5* and *Fgf5*, and ‘neural development’ (FDR=1.3e-7). At these sites, Esrrb and Nr5a2 opposed binding of Oct4 and Sox2, which are also known to induce ESC differentiation when overexpressed (Niwa et al., 2000). These results indicate that Esrrb and Nr5a2, although displaying some specificity in function, tend to cooperate to maintain TF binding at the majority of the regulatory elements they occupy, except at regions where the master pluripotency TFs Oct4 and Sox2 are likely required to facilitate differentiation.

### Loss of pluripotency TF binding leads to major gene expression responses underlying the loss of self-renewal and the transition to early differentiation

**Fig. 4.**
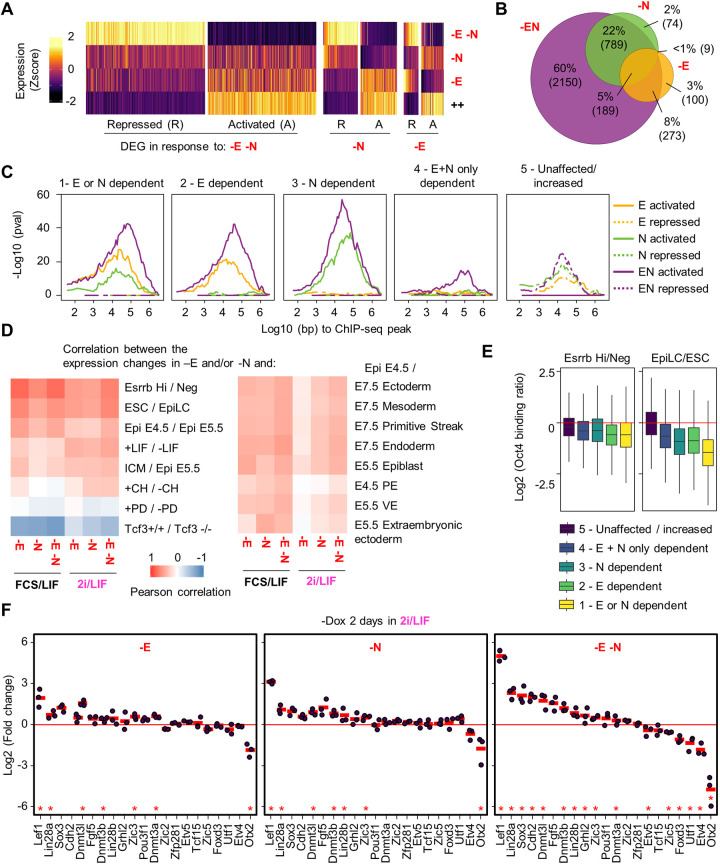
**Esrrb and Nr5a2 control gene expression to restrain differentiation and support naive pluripotency.** (A) Heatmap showing Z-score expression of genes significantly responding (FDR<0.01, absolute fold change>1.5) 2 days after depletion of Esrrb (−E), in the absence of Nr5a2 (−N), and after depleting Esrrb in the absence of Nr5a2 (−E−N), in cells cultured in 2i/LIF. Presented as in Fig. 2D. (B) Venn diagram showing the overlap between genes responding to the depletion of Esrrb (−E), Nr5a2 (−N) or both TFs (−E−N). (C) Statistical enrichment (−log10 hypergeometric right-tail *P*-value) of excess genes responsive to the depletion of Esrrb, Nr5a2, or both TFs as a function of the distance to the different classes of regions conjunctly bound by Esrrb, Oct4, Sox2 and Nanog identified in Fig. 3B. (D) Heatmap displaying the Pearson correlation coefficient between the fold change in gene expression observed in this study after depletion of Esrrb (−E), Nr5a2 (−N) or both TFs (−E−N), and between the indicated conditions in published reports (see Materials and Methods for details). (E) Box plot, presented as in Fig. 1H, showing the fold change in Oct4 binding between EsrrbHi and Esrrb^Negative^ ESCs (as reanalysed from Festuccia et al., 2018a), or between ESC and epiblast-like stem cells (EpiLC; Buecker et al., 2014), at the different classes of Esrrb- and Nr5a2-responsive elements identified in Fig. 3B. (F) RNA-seq fold change of gene expression 2 days after depletion of Esrrb (−E), in the absence of Nr5a2 (−N), and after depleting Esrrb in the absence of Nr5a2 (−E−N). All cells were grown in 2i/LIF; each circle represents an independent experiment (*n*=3); the mean is marked by a red horizontal line. *FDR<0.01.

Next, we sought to determine which genes are deregulated in response to the loss of Esrrb and Nr5a2 in ESCs cultured in 2i/LIF. RNA-seq in EKOiE and EKOiE NrKO before or 2 days after Dox withdrawal identified genes differentially expressed (FDR<0.01) after depletion of Nr5a2 (546 downregulated, 515 upregulated) or Esrrb (343 down, 228 up), and highlighted that the targets of the two factors are largely overlapping (Fig. 4A,B, Table S2). Nonetheless, uniquely responsive genes could also be identified. In line with the pronounced effect on TF binding, loss of both Esrrb and Nr5a2 affected a higher number of genes than the individual depletion of either factor (1662 downregulated, 1779 upregulated). Of note, these transcripts showed less pronounced but concordant changes after individual depletions, confirming that Esrrb and Nr5a2 largely act in concert to control gene expression (Fig. 4A). We then measured the propensity of the five classes of regulatory regions we identified to be enriched in the vicinity of genes responding to Esrrb and Nr5a2 (Fig. 4C). We observed a very strong statistical enrichment of genes activated by the two orphan receptors around clusters 1-3, for which the dual loss of Esrrb and Nr5a2 leads to a strong decrease in Oct4, Sox2 and Nanog. Moreover, genes sensitive to Esrrb or Nr5a2 were also enriched in the vicinity of regions where either Esrrb or Nr5a2, respectively, are required for TF binding. We also observed that regions showing a looser dependency on Esrrb or Nr5a2 displayed lower statistical enrichment around responsive genes. Finally, we noticed that genes repressed by Esrrb and/or Nr5a2 were associated with regions where the two factors tend to oppose Oct4 and Sox2 occupancy. Altogether, this analysis suggests that Esrrb and Nr5a2 act more directly as activators than repressors, and that, to repress gene expression, both TFs often restrain Oct4/Sox2 binding.

We next explored the functional relevance of the gene expression changes mediated by Esrrb and Nr5a2. First, we observed that genes activated by Esrrb and Nr5a2 in 2i/LIF play a role in pluripotency regulation (‘response to LIF signalling’, FDR=1.2e-7) and in energetic and metabolic processes (e.g. ‘dicarboxylic acid metabolic process’, FDR=8.5e-5) (Table S3). Notably, when we compared the effect of the loss of Esrrb and Nr5a2 with the response to LIF stimulation in ESCs (Martello et al., 2013), we observed a correlated response (Fig. 4D, Pearson correlation coefficient 0.47). This prompted us to study more specifically the intersection of the activity of Esrrb/Nr5a2 with that of signalling pathways. In particular, both Esrrb and Nr5a2 have been suggested to act downstream of WNT (Martello et al., 2012; Wagner et al., 2010). We thus set out to compare the effects of the loss of Tcf3 (Yi et al., 2011), the main mediator of WNT activity in ESCs, and that of the two TFs. Genes induced or downregulated in *Tcf3*^−/−^ ESCs were respectively repressed or activated by Esrrb or Nr5a2 (Fig. 4D, Pearson correlation coefficient Esrrb: −0.24; Nr5a2: −0.41). Notably, the concomitant loss of both Esrrb and Nr5a2 resulted in a higher correlation (−0.51). These results suggest that Esrrb and Nr5a2 may act redundantly as mediators of the effects of WNT signalling in ESCs, possibly explaining why the individual deletion of either gene is tolerated in 2i/LIF.

Finally, we focused on genes upregulated after loss of both Esrrb and Nr5a2, and found that they are enriched for Gene Ontology terms linked to cell differentiation and developmental progression (e.g. ‘somitogenesis’ FDR=1.99e-5, ‘embryonic pattern specification’ FDR=5.16e-4; Table S3). In agreement with this, we observed highly concordant transcriptional changes after the depletion of Esrrb and Nr5a2, and during the early stages of ESC differentiation, both when occurring spontaneously in FCS/LIF (Festuccia et al., 2018a) and when directly driven from 2i/LIF towards epiblast-like stem cells (Buecker et al., 2014) (Fig. 4D, Pearson correlation coefficients 0.67, 0.64, respectively). This strongly indicates that Esrrb and Nr5a2 cooperate in restraining the exit from naïve pluripotency. Because early differentiation is accompanied by a reorganisation of Oct4 binding (Buecker et al., 2014; Festuccia et al., 2018a), we compared the changes in Oct4 occupancy observed after the loss of Esrrb and Nr5a2 with those previously reported. Regions showing increasing dependence on the two orphan receptors also displayed a progressively more severe reduction in Oct4 binding, either in differentiating cells or during the conversion to epiblast-like stem cells (Fig. 4E). Reciprocally, enhancers maintaining or gaining Oct4 after Esrrb and Nr5a2 depletion (Group 5) were less affected. These observations suggest that Esrrb and Nr5a2 downregulation, early in the progression of differentiation, may play a causal role in triggering the dismantling of naïve pluripotency, allowing the transition to a formative/primed state (Smith, 2017). In support of this, we detected the upregulation of *Lef1*, *Lin28*, *Fgf5*, Dnmt genes, *Sox3*, *Zic3* and *Grhl2* after the double depletion (Fig. 4F), and an overall correlation between the gene expression changes consequent to the loss of Esrrb/Nr5a2 and those occurring in the epiblast upon implantation (Fig. 4D, Pearson correlation coefficients: left panel Epi E4.5/Epi E5.5: 0.42, ICM/Epi E5.5: 0.36, from Boroviak et al., 2015; right panel Epi E4.5/Epi E5.5 0.36, from Argelaguet et al., 2019). Yet, as recently reported (Carbognin et al., 2020), Esrrb activity not only globally opposes, but also contributes to preparing the rewiring of the pluripotency network: Esrrb appears to license expression of specific regulators of formative/primed pluripotency. Crucially, also in this context Esrrb and Nr5a2 display clear synergy, as shown by the complete silencing of *Otx2*, and the downregulation of *Foxd3*, *Utf1* and *Etv4* following loss of either or, more pronouncedly, both TFs (Fig. 4F). Finally, the gene expression changes triggered by the loss of both nuclear receptors broadly correlated with those observed when comparing the E4.5 epiblast to cells of the three germ layers, at later embryonic stages (Fig. 4D, Pearson correlation coefficients: Epi E4.5/E7.5 Ectoderm 0.39, Epi E4.5/E7.5 Mesoderm 0.35, Epi E4.5/E7.5 Endoderm 0.42, from Argelaguet et al., 2019). Together with the upregulation of a broad panel of differentiation markers (Fig. 3D), these results indicate that the acute depletion of Esrrb and Nr5a2 eventually results in the differentiation towards multiple lineages.

Overall, our analysis establishes that Esrrb and Nr5a2 are required to assist TF binding at most of the regions they occupy: in their absence, the pluripotency network and gene expression programme collapses. In addition, at a subset of developmental genes, Esrrb and Nr5a2 oppose Oct4 and Sox2 binding, possibly attenuating their differentiation-inducing effects. Through these two mechanisms, which are reminiscent of those observed for Nanog (Heurtier et al., 2019), Esrrb and Nr5a2 mediate the fundamental function of maintaining the expression of developmental triggers in check. These results therefore identify Esrrb and Nr5a2 as set of redundant but essential regulators of pluripotency.

## DISCUSSION

Our results identify a functional overlap between two members of the same family of orphan nuclear receptors, Esrrb and Nr5a2, in supporting pluripotency in mouse ESCs. Similar redundant functions have been described for another family of auxiliary pluripotency TFs, KLFs (Di Giammartino et al., 2019; Yamane et al., 2018). The individual deletion of Klf4, Klf2 and Klf5 is tolerated by ESCs, but not the concomitant loss of all three TFs. Although these results present analogies with our findings, experiments were not performed in conditions in which self-renewal is reinforced, as in 2i/LIF. It thus remains to be determined whether KLF factors are strictly required for the maintenance of the undifferentiated state. Intriguingly, not all member of the KLF family are able to support self-renewal, which is explained by variations in their DBD (Yamane et al., 2018). Of note, Nr5a2 and Esrrb are part of a group of orphan nuclear receptors that possess an extension of the DBD beyond the zinc-finger domain, which allows monomeric binding to DNA (Gearhart et al., 2003; Solomon et al., 2005). Consequently, this class of nuclear receptors recognises a common half-palindromic, extended, oestrogen response element. The ability to access similar binding sites at regulatory regions emerges therefore as a requirement for the functional redundancy between TFs.

Although Esrrb and Nr5a2 are able to recognise similar motifs, we report a preference for the presence of either T or C at the seventh base in their consensus binding sequences. Yet, this specificity is nuanced, as supported by biochemical data. First, footprinting experiments show that the seventh base of the motif does not mediate strong interactions with either nuclear receptor (Galarneau et al., 1996; Yang et al., 1996). In addition, Nr5a2 binds to both T or C motif variants in electrophoresis mobility shift assays (Chen et al., 2001; Hinshelwood et al., 2003). Finally, crystal structures are available for both Esrrb and Nr5a2 DBDs bound respectively to T or C containing motifs (Gearhart et al., 2003; Solomon et al., 2005; Weikum et al., 2016). In hNR5A2 and in hESRRB, E104 and K128, respectively, are responsible for establishing specific contacts with the seventh base: these residues are conserved in mouse and do not vary between Esrrb and Nr5a2. Thus, the binding preference of Esrrb and Nr5a2 does not result from a divergence in the residues directly contacting the discriminating base, but may depend on the global organisation of the DBDs, possibly justifying a relaxed specificity. Despite being mild, these preferences are of functional significance: regions in which one or the other motif variant is more represented respond preferentially to the depletion of the favoured TF.

Our data show that Esrrb and Nr5a2 can bind DNA independently, but cooperate to access their targets: in particular, the depletion of Esrrb attenuates Nr5a2 binding at many enhancers, including at regions where the two TFs recognise the same motifs. The reciprocal dependence is globally less prominent, which might be explained by the lower expression of Nr5a2. Of note, whereas the presence of an exact match to the Nr5a2 consensus is highly enriched at regions showing prevalent Nr5a2 binding, perfect Esrrb motifs preferentially appear in regions showing balanced occupancy by the two TFs. This may indicate that DNA binding by the less abundant Nr5a2 molecules is either driven by the presence of a strong motif, or facilitated by Esrrb occupancy. In support, in the absence of Esrrb, the quality of Nr5a2-binding sites becomes a better predictor of its occupancy levels. Irrespective of the magnitude of these reciprocal effects, the ability of Esrrb and Nr5a2 to cooperate at the very same locations has important implications. Taken together with the high structural similarity of the two TFs, it establishes that the functional overlap between Esrrb and Nr5a2 goes beyond, and is conceptually distinct from, the generically additive activity displayed by many pluripotency regulators (Dunn et al., 2014). Unrelated TFs access binding sites that are broadly distributed over regulatory regions, which leads to a variety of functional interactions that are dependent on the grammar of motifs present at these loci, and on effects mediated by the chromatin and recruited co-factors. Esrrb and Nr5a2 act instead at a common set of motifs, exerting a clearly concordant action.

Of further relevance to the mechanism mediating cooperativity between the two TFs, it is noteworthy that orphan nuclear receptors are able to bind DNA not only as monomers, but also as homodimers or heterodimers (Huppunen and Aarnisalo, 2004; Razzaque et al., 2004). Thus, in principle, Esrrb and Nr5a2 could access their targets in complex. Finally, at least two other classes of orphan receptors recognising extended half-sites – Nr1d1 and Nr1d2 (Rev-Erb alpha and beta) and Nr4a1 (NgfIb) (Harding and Lazar, 1993; Wilson et al., 1992) – are expressed in ESCs at levels comparable to Nr5a2. The interplay of these TFs, and Esrrb/Nr5a2 in supporting pluripotency deserves to be investigated in depth.

Our results, and the proposal that the function of highly related TFs should not be studied in isolation, question which TFs should be considered as ‘core’ regulators of pluripotency. Oct4 and Sox2 are prominent amongst other pluripotency TFs, in that they perform essential functions both during development and in ESCs (Avilion et al., 2003; Nichols et al., 1998). Another TF, Nanog, is required for epiblast specification (Mitsui et al., 2003; Silva et al., 2009), but is not essential in ESCs, where it fine-tunes rather than enables self-renewal (Chambers et al., 2003, 2007). Here, we show that Esrrb and Nr5a2 collectively play an essential role in ESCs. Although detrimental, the loss of either factor alone does not fully compromise the maintenance of pluripotency (Adachi et al., 2018; Atlasi et al., 2019; Festuccia et al., 2012; Fujii et al., 2015; Gu et al., 2005; Martello et al., 2012; Sladitschek and Neveu, 2019). In line with this, neither the loss of Esrrb, nor that of Nr5a2, results in developmental defects before implantation (Gu et al., 2005; Labelle-Dumais et al., 2006; Luo et al., 1997). Esrrb deletion leads to defective placental development, and developmental arrest around E9.5, whereas Nr5a2 ablation results in gastrulation defects, and a severe phenotype emerges between E6.5 and E7.5. In light of our results in ESCs, it will be now important to determine the effect of the concomitant loss of Esrrb and Nr5a2 during early development. A clear requirement for the establishment of pluripotency would elevate the two orphan receptors on equal terms with Oct4 and Sox2. Even then, whereas Esrrb and Nr5a2 are downregulated upon implantation, Oct4 and Sox2 continue to play an essential role in primed pluripotent cells (Brons et al., 2007; Mulas et al., 2018; Osorno et al., 2012; Tesar et al., 2007). Our results could thus call for a distinction between core naïve activities, and global regulators of pluripotency.

More directly, our results suggest a potential role for Esrrb and Nr5a2 in opposing the premature extinction of pluripotency in the naïve epiblast. Indeed, we show a global correlation between the changes in gene expression and TF binding triggered by the loss of Esrrb and Nr5a2 and those observed during the conversion between naïve and primed pluripotency, both in culture and during development. This confirms previous results highlighting how, during the early stages of ESC differentiation, the spontaneous downregulation of Esrrb instates a transcriptional state that displays analogies to primed pluripotency (Festuccia et al., 2018a). Importantly, extending previous reports (Carbognin et al., 2020), we find that Esrrb and Nr5a2 sustain expression of key primed pluripotency regulators, in particular Otx2, Foxd3 and Etv4. Although the activity of several of these factors have been proposed to play a determining role in rewiring the pluripotency network during the dismantling of naïve pluripotency (Buecker et al., 2014; Chen et al., 2018; Respuela et al., 2016; Yang et al., 2014), the global transcriptional changes we observe despite their attenuated expression suggest that the loss of naïve TFs, and thus of cooperative interactions, plays an equally determinant role in driving the transition between pluripotent states.

Our results contribute to the construction of a mechanistic framework that will further our understanding of how the mutual dependency between single components of the pluripotency network underlies both its robustness and its ability to respond to differentiation cues. Multiple mechanisms of cooperativity between pluripotency TFs have been documented that likely contribute to these functional interactions: from the binding of Oct4 and Sox2 to DNA as an heterodimer (Remenyi et al., 2003; Tapia et al., 2015; Yuan et al., 1995), to the recognition by different members of the same class of TFs of related sequences – as for KLFs (Yamane et al., 2018) or Esrrb/Nr5a2 – to more indirect interactions mediated by the chromatin. In particular, Oct4 or Nanog have been reported to instate accessibility at target enhancers to foster TF binding (Heurtier et al., 2019; King and Klose, 2017). It will be now important to determine how chromatin remodellers, in particular SWI/SNF and NuRD complexes, which interact with Esrrb in ESCs (van den Berg et al., 2010), mediate the molecular activities of Esrrb and Nr5a2. Indeed, the ability to reposition or displace nucleosomes might underlie part of the effects we observe. Short distances between Esrrb/Nr5a2 and Oct4/Sox2 motifs – within the range covered by a nucleosome - are prevalent at regions where the binding of other pluripotency factors is dependent on the two nuclear receptors. It remains to be understood in finer detail how the relative arrangement of TF-binding sites at these enhancers determines their functional responses.

Altogether, we report that Esrrb and Nr5a2 represent two arms of a single functional module that is at the top of the hierarchy of gene regulation in ESCs. This notion opens new research avenues in developmental and stem cell biology. In particular, the collective contribution of orphan nuclear receptors to the maintenance of pluripotency in human ESCs, especially in the less-characterised naïve state, needs further attention. By analogy to the functional overlap described between Esrra and Esrrg during somatic cell reprogramming and heart development (Dufour et al., 2007; Kida et al., 2015), it is tempting to speculate that different combinations of nuclear orphan receptors may play a conserved role in pluripotency and during mammalian embryogenesis.

## MATERIALS AND METHODS

### General culture conditions

ESCs were cultured on 0.1% gelatine (Sigma-Aldrich, G1890-100G) in DMEM+GlutaMax-I (Gibco, 31966-021), 10% FCS (Gibco, 10270-098), 100 μM 2-mercaptoethanol (Gibco, 31350-010), 1× MEM non-essential amino acids (Gibco, 1140-035) and 10 ng×ml^−1^ recombinant LIF (Miltenyi Biotec, 130-099-895). When indicated, cells were grown in 2i-containing medium (1 µM PD0325901 and 3 µM CHIR99021; Axon Medchem): 0.5× DMEM/F12 (Gibco, 31331093), 0.5×Neurobasal (Gibco, 21103049), 0.5% N2 supplement 100× (Gibco, 17502048), 1% B27 supplement 50× (Gibco, 17504044), 10 µg/ml insulin (Sigma-Aldrich, I1882-100MG), 2 mM L-glutamine (Invitrogen, 91139), 0.05% BSA (Sigma-Aldrich, A3311-10G), 100 μM 2-mercaptoethanol (Gibco, 31350-010), 10 ng/ml recombinant LIF (Miltenyi Biotec, 130-099-895).

### Derivation of EKOiE NrKO and EKOiE NrKO repair ESCs

The fourth exon of *Nr5a2* (Nr5a2-205), encoding the first portion of the DNA-binding domain, was disrupted in EKOiE ESCs (Festuccia et al., 2016). Two clones bearing deletions on both alleles were selected for further experiments (c2 and c4). In Clone 4 EKOiE NrKO ESCs, one of the two *Nr5a2* alleles was repaired using a template obtained by PCR from E14Tg2a genomic DNA. Further details on the derivation of EKOiE NrKO ESCs and of all other lines used in this study are detailed in the supplementary Materials and Methods.

### Immunofluorescence, live-cell imaging, and alkaline phosphatase staining

Cells were plated on Ibidi hitreat plates coated overnight with 0.01% poly-L-ornithine (Sigma-Aldrich, P4957) at 4°C, washed and coated for 2 h with 10 μg/ml laminin (Millipore, CC095) in PBS. Fixation was performed for 10 min in 1% formaldehyde (Thermo Scientific, 28908) at room temperature (RT). After washing the cells twice in PBS, they were permeabilised with 0.1% v/v Triton X-100 in PBS (PBSTx) supplemented with 3% donkey serum (Sigma-Aldrich, D9663) for 30 min at RT. Primary antibodies (diluted in PBSTx with 3% donkey serum) were applied for 2 h at RT or overnight at 4°C in a volume of 1 ml per dish. After three washes in PBSTx, secondary antibodies (2 μg/ml in PBSTx with 3% donkey serum) were applied for 2h at RT. Cells were washed three times in PBSTx, nuclei counterstained with 4',6-diamidino-2-phenylindole (DAPI; Sigma-Aldrich, D9542), and imaged with an inverted Leica SP8 confocal microscope using a 40× oil immersion objective. Acquisition was performed using the LASX acquisition software suite. Primary antibodies used were: 0.3 μg/ml anti-Nanog rabbit polyclonal (Cosmo Bio, REC-RCAB001P); 0.4 μg/ml anti-Oct4 mouse monoclonal - clone C10 (Santa Cruz Biotechnology, sc-5279), for staining in combination with anti-Sox2; 1 µg/ml anti-Oct4 rabbit polyclonal (Abcam, ab19857), for staining in combination with anti-Flag; 1:500 anti-Sox2 rabbit polyclonal (Active Motif, 39843); 1 µg/ml anti-Klf4 goat polyclonal (R&D Systems, AF3158); 1 μg/ml anti-Flag mouse monoclonal (Sigma-Aldrich, F3165). Secondary antibodies used were: Alexa Fluor 594 AffiniPure Donkey Anti-Rabbit IgG (H+L) (Jackson ImmunoResearch, 711-585-152); Alexa Fluor 488 AffiniPure Donkey Anti-Mouse IgG (H+L) (Jackson ImmunoResearch, 715-545-150); Alexa Fluor 647 AffiniPure Donkey Anti-Goat IgG (H+L) (Jackson ImmunoResearch, 705-605-147).

For live-cell imaging, Nr5a2-GFP+Esrrb-mCherry ESCs grown in FCS/LIF or 2i/LIF in Ibidi plates, as described above, were incubated with 500 nM Hoechst-33342 for 20 min before imaging. During imaging, the cells were kept at 37°C in a humidified atmosphere (7% CO_2_). Images on single focal planes were acquired with a 63× oil immersion objective on an inverted LSM800 confocal Zeiss microscope, using the ZEN Blue acquisition software suite.

For alkaline phosphatase staining, 300 EKOiE, EKOiE Nr5a2 KO (c2 and c4), and EKOiE Nr5a2 KO Repair ESCs (c4.2 and c16.1) were plated in single wells of 6-well plates coated overnight with 0.01% poly-L-ornithine (Sigma-Aldrich,P4957) at 4°C, washed and coated for 2 h with 10 μg/ml laminin (Millipore, CC095) in PBS. After culture in FCS/LIF or 2i/LIF media in the presence or absence of 1 μg/ml doxycycline (Sigma-Aldrich, I5148) for 7 days, cells were fixed and stained using an alkaline phosphatase staining kit (Sigma-Aldrich, 86R-1KT) according to the manufacturer's instructions. Representative images of alkaline phosphatase-stained colonies formed by EKOiE, EKOiE Nr5a2 KO and EKOiE Nr5a2 KO repair ESCs were acquired using a Zeiss Discovery V8 Stereo microscope, and the ZEN blue software suite.

### Growth curves

0.75×10^6^ EKOiE or EKOiE Nr5a2 KO ESCs were plated in single wells of 6-well plates and passaged every 2 days in the absence or presence of doxycycline, replating each time 0.75×10^6^ cells. At each passage, cells were set aside for RNA extraction.

### Flow cytometry

E14Tg2a, Esrrb-T2a-GFP or Nr5a2-T2a-GFP ESCs were plated at low density (2000 cells/cm^2^) in FCS/LIF medium and cultured for 3 days before analysis. After trypsinisation, cells were resuspended in FCS/LIF without Phenol Red, and analysed using a LSR II flow cytometer system or a Luminex Image Stream MK2 instrument with a 60× magnification objective. Data were analysed using the FlowJo software suite.

### Chromatin immunoprecipitation (ChIP)

TF binding was assessed in E14Tg2a Flag-Nr5a2 ESCs cultured in FCS/LIF, and EKOiE, EKOiE NrKO or EKOiE HA-AID-Nr5a2 ESCs cultured in 2i/LIF. Approximately 10^7^ ESCs were crosslinked in 2 ml of PBS-DSG 2 mM (Sigma-Aldrich, 80424-5mg) for 50 min at followed by 10 min in 1% formaldehyde PBS (Thermo Scientific, 28908). Chromatin preparation, immunoprecipitations and library preparations are described in the supplementary Materials and Methods. Antibodies: anti-Esrrb mouse monoclonal (1 µg per 2×10^6^cells, Perseus Proteomics, H6-705-00), anti-Nanog rabbit polyclonal (0.6 μg per 2×10^6^cells, Cosmobio, REC-RCAB001P); anti-Oct4 rabbit polyclonal (1 μg per 2×10^6^cells, Abcam, ab19857); anti-Sox2 rabbit polyclonal (1 μl per 2×10^6^cells – concentration not specified, Active Motif,39844); anti-Flag mouse monoclonal (1 μg per 2×10^6^cells Sigma-Aldrich, F3165); anti-HA mouse monoclonal (1 μg per 2×10^6^cells, clone 12CA5 – Roche, 11 583 816 001).

### Gene expression analysis by RNA-seq

2×10^5^ EKOiE or EKOiE NrKO ESCs were cultured in wells of 6-well plates with/without 1 µg/ml doxycycline (Sigma-Aldrich, I5148) for 2 days before RNA extraction with 500 μl TRIzol (Thermo Fisher, 15596026) according to the manufacturer's instructions. To eliminate any genomic DNA contamination, this was followed by an additional DNAse I treatment (Qiagen, 79254) for 20 min at 37°C followed by phenol:chloroform purification. RNAs were resuspended in Ultrapure DNAse/RNase Free Distilled Water (Fisher Scientific, 10977035). Stranded, poly-A selected RNA-seq libraries were prepared and sequenced (paired-end 150bp reads) by Novogene.

### Gene expression analysis by RT-qPCR

RNA was prepared with a Nucleospin RNA kit, performing DNAse I treatment (Macherey-Nagel, 740955.50). One microgram of RNA was used for cDNA preparation using a Transcriptor First Strand cDNA synthesis kit (Roche, 04897030001), using random hexamer priming. Real-time RT-PCR reactions were performed in triplicate in 384-well plates with a 480 LightCycler (Roche) using the LightCycler 480 SYBR Green I Master mix (Roche, 04887352001. PCR primer sequences and full details are given in the supplementary Materials and Methods.

### Western blot

10^6^ cells were lysed in 100 µl RIPA buffer with 1× protease inhibitor cocktail (Roche, 04693116001) and incubated for 2 h with 2500 U/ml benzonase (Sigma-Aldrich, E1014) at 4°C. Samples were boiled in Laemmli Buffer (Bio-Rad, 161-0737), and 20 µl of each sample was loaded onto 10% Mini-PROTEAN TGX gels (Bio-Rad, 4568033) run in glycine/SDS buffer and transferred onto a nitrocellulose membrane (Amersham, 10600003), which was blocked, and incubated overnight with primary antibodies in PBSTw (0.1% Tween-20 in PBS) with 5% BSA, washed and incubated with secondary antibodies. Membranes were then washed in PBSTw and, where relevant, incubated with PIERCE ECL2 Western Blotting Substrate (Thermo Scientific, 80196) before detection. A list of primary and secondary antibodies used, and a detailed description of the method is available in the supplementary Materials and Methods.

### Computational methods

Full details on the computational methods used in this study are available in the supplementary Materials and Methods.

#### ChIP-seq

ChIP-seq was performed in triplicate, except for duplicate Nr5a2 binding profiles in 2i/LIF. Paired-end reads were trimmed, aligned with Bowtie2 (Langmead and Salzberg, 2012) to the mm10 genome, filtered for ‘single discovered’ and edit distance <4. Duplicate reads were collapsed. Peaks were called against inputs using MACS2 (Feng et al., 2012) and intersection with the mm10 blacklist (Encode_Project_Consortium, 2012) excluded. For defining Nr5a2- or Esrrb-bound regions in FCS/LIF, we required that a peak must be called in all three replicates for either Esrrb or Nr5a2. In 2i/LIF, for each TF we required that a peak must be called in all replicates of a given condition, with the exception of Nr5a2 (one out of two replicates). We then merged the peaks of each TF analysed in FCS/L or 2i/L, respectively, to obtain regions were multiple TF bind. To determine the number of clusters to use, we relied on the enrichment of differentially expressed genes in proximity of ChIP-seq peaks, and found that k=5 was the first k at which enrichment was robust. Differential binding analysis was performed using DESeq2 (Love et al., 2014). Gene Ontology analyses were made with GREAT using standard parameters. *De novo* motif discovery on Esrrb/Nr5a2-bound regions in FCS/L used the Regulatory Sequence Analysis Tools (RSAT) with standard parameters (rsat.sb-roscoff.fr) (Nguyen et al., 2018). To locate motifs preferentially bound by Esrrb and Nr5a2, two PFM matrixes were created to reflect a perfect match to the consensus sequence TCAAGGTCA or TCAAGGCCA, and motifs including 0 or 1 mismatches identified with the TFBSTools R package (Tan and Lenhard, 2016). Alternatively, a motif corresponding to the Jaspar Nr5a2 motif MA0505.1 was trimmed to the consensus TCAAGG**X**CA, leaving complete freedom at the seventh base (bold and underlined). Motif occurrences were identified and the frequency of T or C at the seventh base determined. To calculate enrichments of Oct/Sox motifs (MA0142.1) at a given distance from Esrrb/Nr5a2 motifs, we took all motifs with a score greater than the median and calculated the absolute distance between all pairs of Esrrb and Oct4/Sox2 motifs over the regions. We then compared the observations to a randomised background to derive a Poisson *P*-value for the observed number of pairs at each distance.

#### RNA-seq

RNA-seq in FCS/L was performed in duplicate; RNA-seq in 2i/L in triplicate. Stranded paired end RNA-seq reads were aligned to the mm10 genome using STAR (Dobin et al., 2013) and quantified by RSEM (Li and Dewey, 2011) using the RSEM-STAR pipeline, with additional options ‘--seed 1618 --calc-pme --calc-ci --estimate-rspd --paired-end’. RSEM estimated read counts per sample were rounded for use with DESeq2 (Love et al., 2014). Genes with at least 20 raw counts in all replicates of at least one condition were considered for differential expression analysis. For all differential expression tests, DESeq2 was run without independent filtering; genes considered with absolute FC>1.5 and FDR<0.01 were considered as differentially expressed. Gene Ontology analyses were carried out in PANTHER (geneontology.org) with standard parameters. To determine enrichments of each group of differentially expressed genes in proximity to the ChIP-seq clusters, we calculated hypergeometic right tail *P*-values for the association between differentially expressed genes within *x* bp of a ChIP-seq peak belonging to a cluster, compared with a background of all genes within *x* bp of a cluster peak, for *x* in [1, 1e+6] bp, using the Julia package ProximityEnrichment.jl (https://github.com/owensnick/ProximityEnrichment.jl). Data visualisation was made in R using ggplot2 (Wickham, 2016) and ComplexHeatmaps (Gu et al., 2016b) packages.

#### Comparisons with published datasets

Oct4 ChIP-seq datasets from Buecker et al. (2014) and Festuccia et al. (2018a) were obtained through the European Nucleotide Archive database and aligned with Bowtie2 (Langmead and Salzberg, 2012) to the mm10 genome, with default options. Coverage in the classes of Esrrb/Nr5a2-dependent or -independent regulatory regions identified in this study was quantified in each external dataset using the R packages bamsignals (Mammana and Helmuth, 2021), Rsamtools (Morgan et al., 2021) and GenomicRanges (Lawrence et al., 2009). Processed data for RNA-seq correlations were obtained from supplementary tables available in the previously published studies (Argelaguet et al., 2019; Boroviak et al., 2015; Buecker et al., 2014; Dunn et al., 2019; Festuccia et al., 2018a; Martello et al., 2013; Ye et al., 2013; Yi et al., 2011). Focusing on differentially expressed genes, Pearson correlation coefficients were then calculated based on the fold changes in expression of each gene in the external datasets, compared with the fold change in expression observed after depletion of Esrrb, Nr5a2 or both Esrrb and Nr5a2 in FCS/LIF or 2i/LIF. Heatmaps displaying the correlation between datasets were generated using the ComplexHeatmap R package (Gu et al., 2016b). Processed data for RNA-seq analysis of Esrrb and Nr5a2 relative levels of expression was obtained from supplementary tables available in the previously published studies (Atlasi et al., 2019; Buecker et al., 2014; Cruz-Molina et al., 2017; Dunn et al., 2019; Ficz et al., 2013; Gu et al., 2016a; Hsu et al., 2019; von Meyenn et al., 2016). Processed single cell RNA-seq (Kolodziejczyk et al., 2015) was obtained from the ESpresso database (https://espresso.teichlab.sanger.ac.uk/), and data plotted with the FlowJo software suite. Data from Buecker et al. (2014), Festuccia et al. (2018a) and Heurtier et al. (2019) are available in Gene Expression Omnibus (GEO) under accession numbers GSE118898, GSE118907 and GSE56138, respectively.

## Supplementary Material

Supplementary information

Reviewer comments
