# Supplementary material for: The combined action of Esrrb and Nr5a2 is essential for murine naïve pluripotency
Source: Development. 2021 Sep 10;148(17):dev199604. doi: 10.1242/dev.199604 (PMC8451941; doi:10.1242/dev.199604)
Supplement: Supplementary information [file develop-148-199604-s1.pdf]

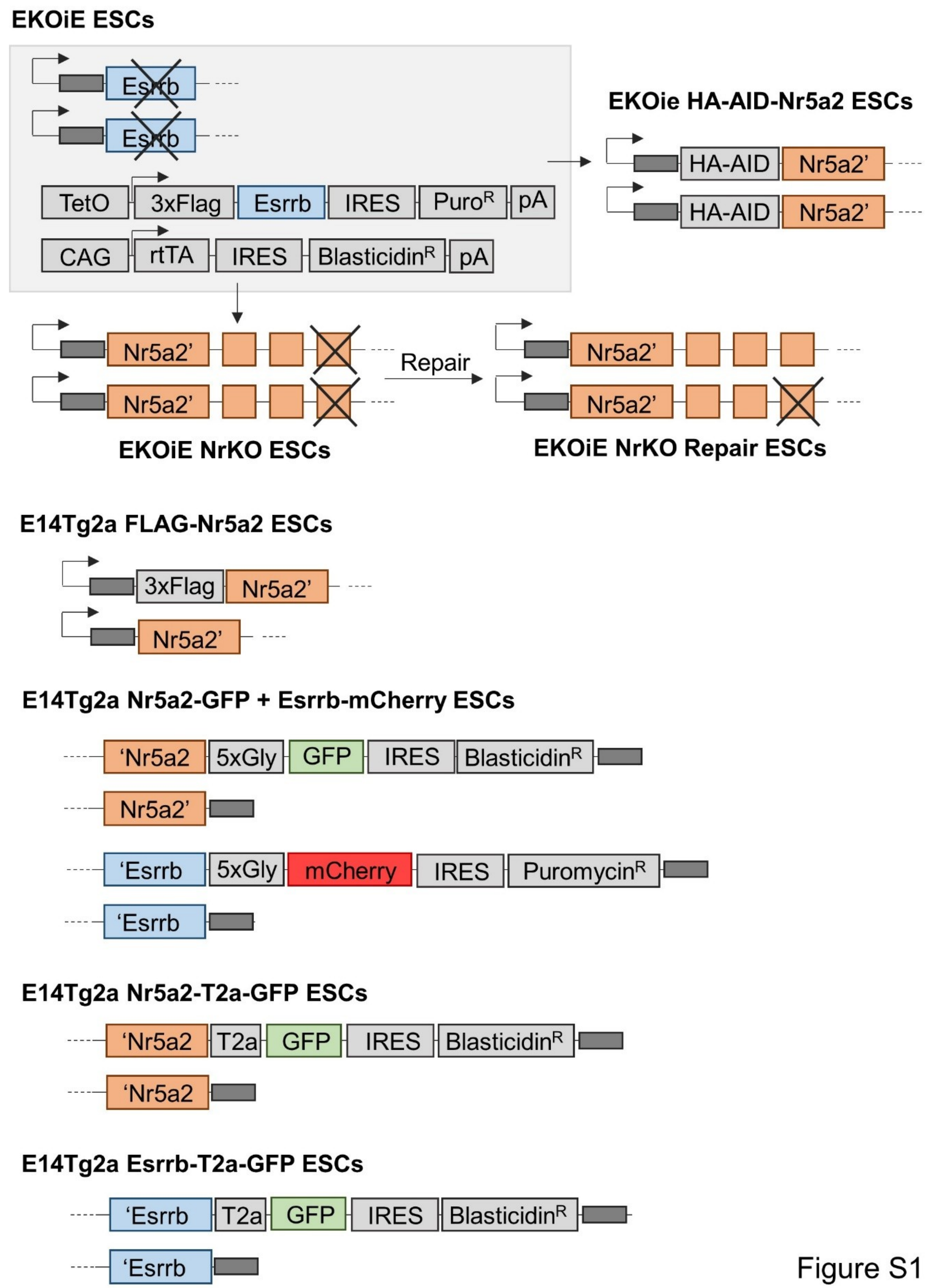


**Fig. S1. Cell lines used in this study.**

Development • Supplementary information

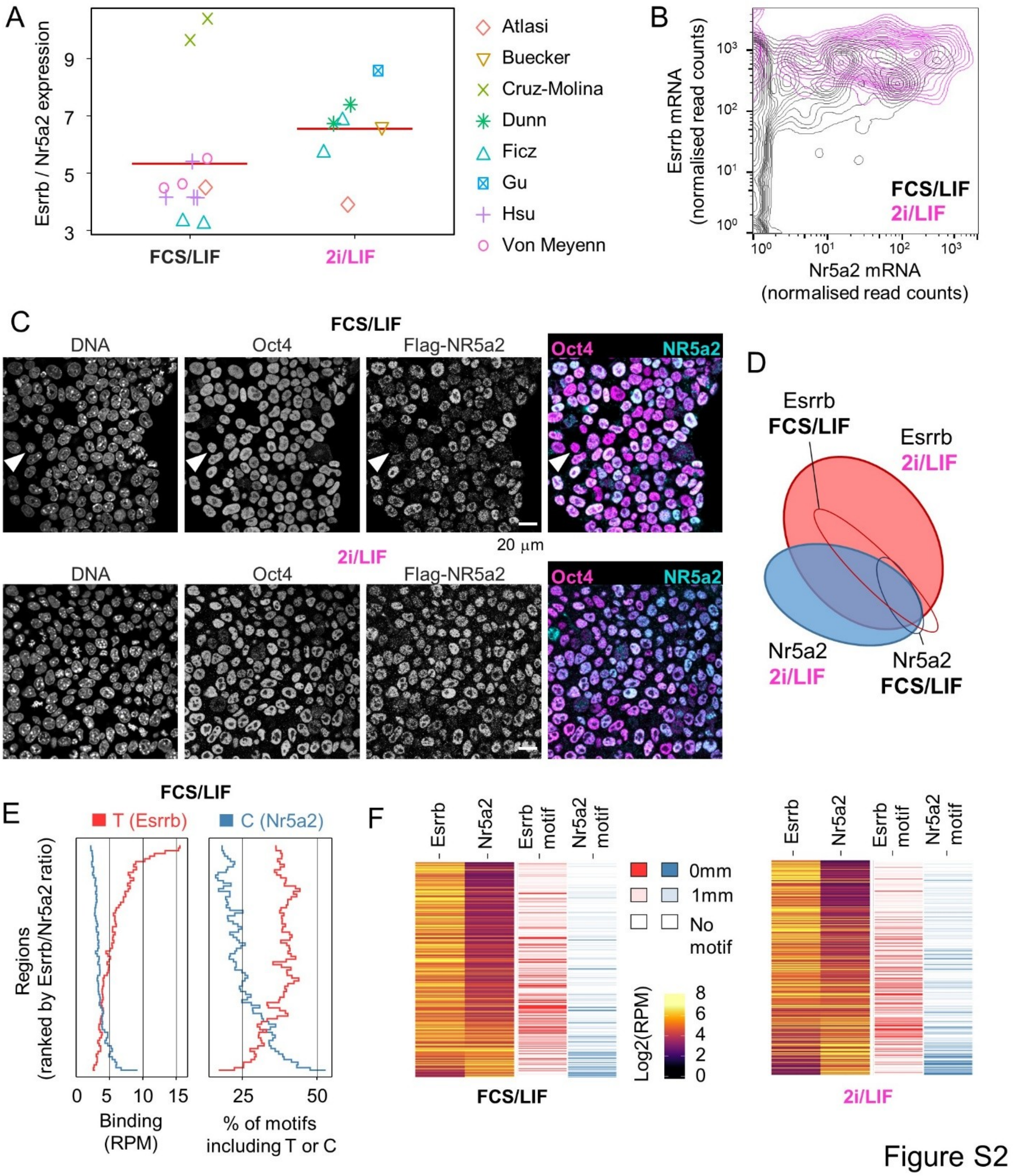


Figure S2

Development • Supplementary information

**Fig. S2. Additional information on Esrrb and Nr5a2 expression and binding.** **(A)** Plot indicating the ratio between Esrrb and Nr5a2 gene expression levels in RNA-seq datasets from the indicated studies (See methods for details). Each dot represents an independent experiment (FCS/LIF n=12, 2i/LIF n=7), the red bars indicate the mean of values in each condition (FCS/LIF or 2i/LIF). **(B)** Density plot showing the distribution of Esrrb and Nr5a2 mRNA levels (normalized read counts) detected in single ESC cells cultured in FCS/LIF or 2i/LIF (data from Kolodziejczyk et al. Cell Stem Cell, 2015). **(C)** Confocal microscopy images showing Oct4 and Nr5a2 expression detected by immunofluorescence in FLAG-Nr5a2 ESC cultured in FCS/LIF or 2i/LIF. Note that Nr5a2 negative cells are prevalently observed in FCS/LIF (white arrowheads). Representative of 2 independent experiments. **(D)** Venn diagram showing the overlap between regions bound by either Esrrb or Nr5a2 in FCS/LIF and 2i/LIF. **(E)** Frequency of motifs including T or C at the 7$^{th}$ position (right) at target regions in FCS/LIF ordered by decreasing Esrrb/Nr5a2 ratio (left, reads Per Million – RPM). **(F)** Heatmaps presenting Esrrb and Nr5a2 occupancy (Reads Per Ten Million – RPM) at target regions ordered by decreasing Esrrb/Nr5a2 ratio (left of each panel) in FCS/LIF or 2i/LIF, along with the occurrence of the Esrrb or Nr5a2 motif as a function of the number of mismatches (mm; right of each panel).

Development • Supplementary information

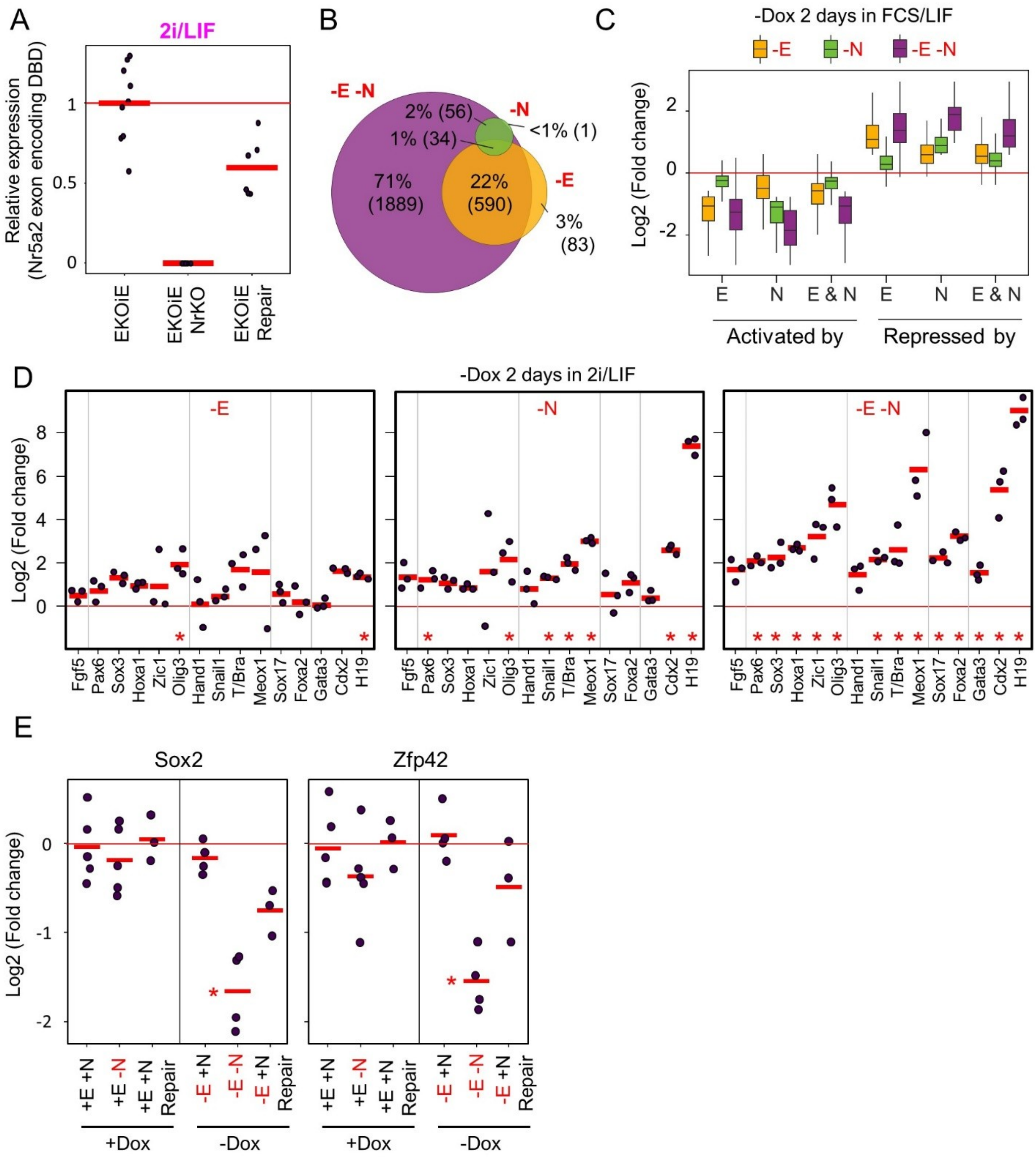


Figure S3

Development • Supplementary information

**Fig. S3. Additional information on the effect of the depletion of Esrrb and/ or Nr5a2 on gene expression. (A)** Relative Nr5a2 expression determined by qPCR using primers annealing to the targeted exon encoding for the DNA binding domain, in EKOiE, EKOiE NrKO ESCs and EKOiE NrKO Repair ESCs cultured in 2i/LIF. Each dot represents an independent experiment (n=9, 9, 6 respectively), the mean is marked by a red horizontal line. **(B)** Venn diagram showing the overlap between genes differentially expressed 2 days after the depletion of Esrrb (-E), in the absence of Nr5a2 (-N), or after depletion of Esrrb in the absence of Nr5a2 (-E-N). **(C)** Boxplot showing the fold change of gene expression of Esrrb, Nr5a2 and Esrrb & Nr5a2 responsive genes two days after inducing the loss of Esrrb, Nr5a2, or both TFs (-E, -N, -E-N as in Fig S3B). The central lines correspond to the median, boxes span from the first to the third quartiles, and whiskers extend to the furthest data point within 1.5xIQR from the boxes. All cells were grown in FCS/LIF. **(D)** RNA-seq fold change of gene expression two days after inducing the loss of Esrrb, Nr5a2, or both TFs (-E, -N, -E-N as in Fig S3B). All cells were grown in 2i/LIF; each dot represents an independent experiment (n=3), the mean is marked by a red horizontal line. * FDR ≤ 0.01. **(E)** Fold change of gene expression determined by qPCR in EKOiE, EKOiE NrKO ESCs and EKOiE NrKO Repair ESCs before (+E+N, +E-N,+E+N repair) or 2 days after (-E+N, -E-N, -E+N repair) withdrawal of doxycycline. All cells were grown in 2i/LIF; each dot represents an independent experiment (n=5,5,3,4,4,3 respectively), the mean is marked by a red horizontal line. * Student's T-test ≤ 0.01 (One-tailed, assuming equal variance, testing each condition against +E+N).

Development • Supplementary information

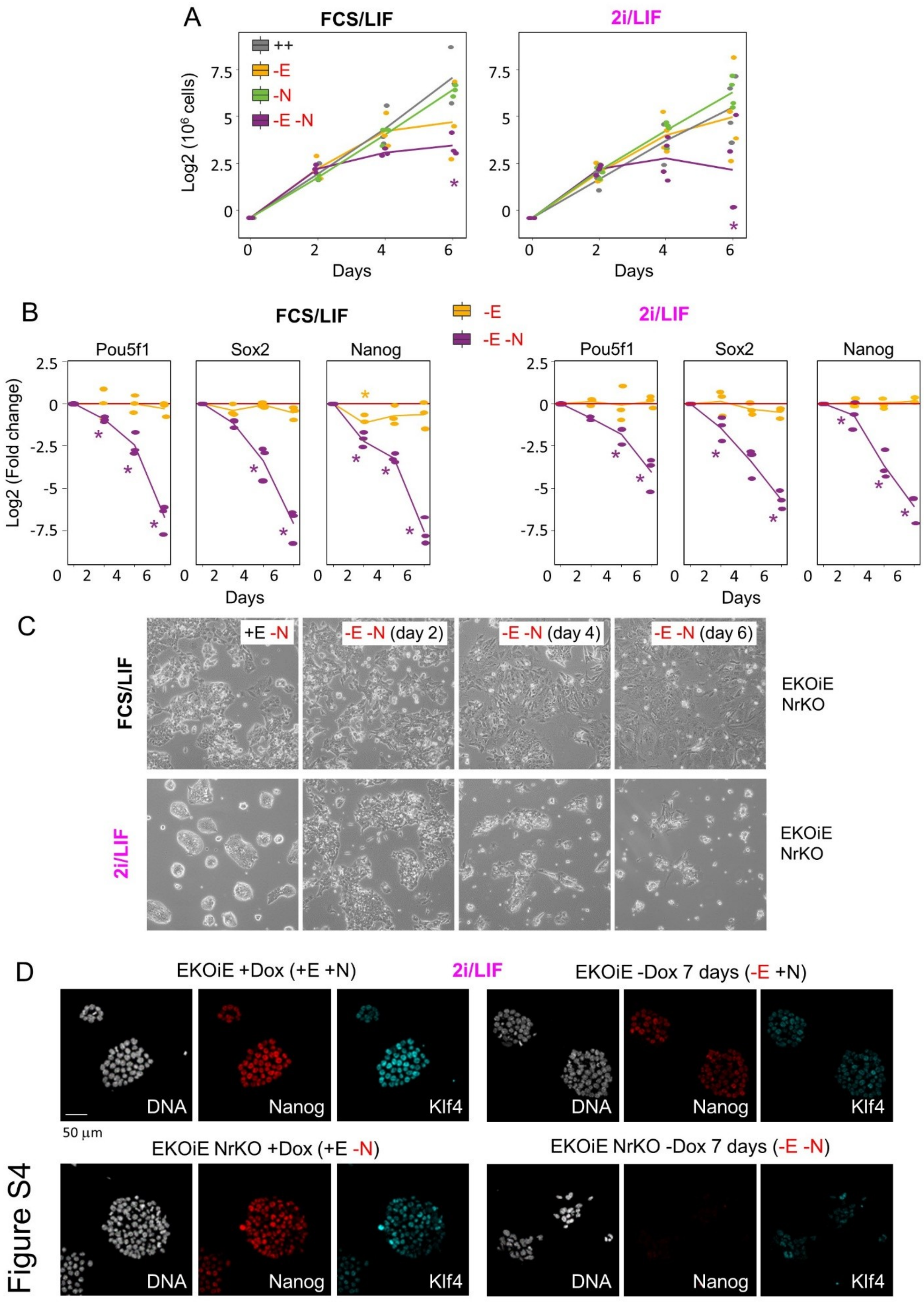
A
FCS/LIF
2i/LIF
++
-E
-N
-E -N
Log2 (10^6 cells)
Days
B
FCS/LIF
2i/LIF
Pou5f1
Sox2
Nanog
Log2 (Fold change)
Days
C
+E -N
-E -N (day 2)
-E -N (day 4)
-E -N (day 6)
FCS/LIF
2i/LIF
EKOiE NrKO
D
EKOiE +Dox (+E +N)
2i/LIF
EKOiE -Dox 7 days (-E +N)
EKOiE NrKO +Dox (+E -N)
EKOiE NrKO -Dox 7 days (-E -N)
DNA
Nanog
Klf4
50 μm

Figure S4

**Fig. S4. Additional information on the effect of the depletion of Esrrb and/ or Nr5a2 on the self-renewal of ESCs.** **(A)** Cumulative cell numbers of EKOiE or EKOiE NrKO ESCs cultured in the presence (++, -N) or absence of doxycycline (-E, -E-N respectively) in FCS/LIF or 2i/LIF, passaging cells every 2 days for the indicated time. Each dot represents an independent experiment (FCS/LIF n=3, 2i/LIF n=4), the mean of each condition at a given time-point is indicated by the colored lines. * Student's T-test ≤ 0.1 (One-tailed, assuming equal variance, testing each condition against ++ at the same time-point). **(B)** Boxplots showing the fold change of gene expression of pluripotency genes in EKOiE ESCs or in EKOiE NrKO ESCs after withdrawal of doxycycline (-E, -E-N respectively) for the indicated time, compared to the each cell line cultured in the presence of doxycycline (time 0). RNA was extracted from the same cells used to derive the growth curves shown in panel A. Each dot represents an independent experiment (n=3), the mean of each condition at a given time-point is indicated by the colored lines. * Student's T-test ≤ 0.01 (One-tailed, assuming equal variance, testing each condition against time 0). **(C)** Images of EKOiE NrKO ESC cultures before and after withdrawal of doxycycline (+E-N, -E-N respectively) for the indicated time. Representative images of the cultures analysed in panel A (n=3, 4). **(D)** Confocal microscopy images showing Nanog and Klf4 expression detected by immunofluorescence in EKOiE or EKOiE NrKO ESCs cultured for 7 days in the presence (+E+N, +E-N) or absence of doxycycline (-E+N, -E-N respectively). Representative of 2 independent experiments.

Development • Supplementary information

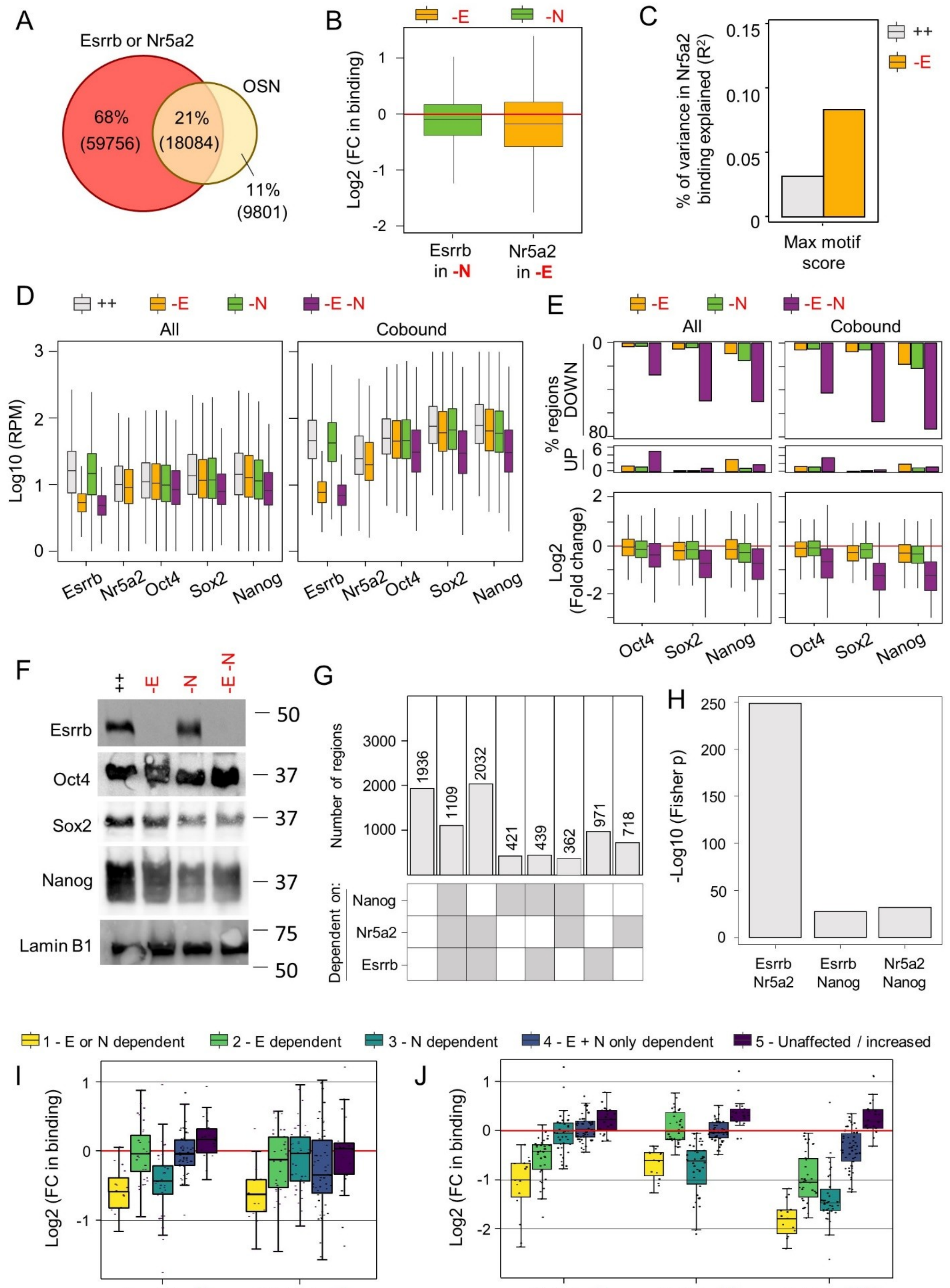


Figure S5

Development • Supplementary information

**Fig. S5. Additional information on pluripotency TF binding after depletion of Esrrb and/or Nr5a2.** **(A)** Venn diagram showing the intersection between regions called as bound by Esrrb or Nr5a2 and by Oct4, Sox2 and Nanog conjunctly (see Methods for details). **(B)** Boxplot showing the fold change in Esrrb and Nr5a2 binding at target regions, 2 days after the depletion of Esrrb (-E) and in the absence of Nr5a2 (-N). The central lines correspond to the median, boxes span from the first to the third quartiles, and whiskers extend to the furthest data point within 1.5xIQR from the boxes. **(C)** Percent of variance in Nr5a2 binding explained (R-squared) by linear regression on the score of the best motif present at regulatory regions, before (++), or 2 days after Esrrb depletion (-E). **(D)** Boxplots, presented as in Fig S5B, showing Esrrb, Nr5a2, Oct4, Sox2 and Nanog binding (Reads Per Ten Million – RPM) at regions bound by any of the TFs, or at regions bound by Oct4, Sox2 and Nanog in conjunction with Esrrb or Nr5a2, in EKOiE ESCs or in EKOiE NrKO ESCs before (++, -N) or 2 days after withdrawal of doxycycline (-E, and -E-N respectively).**(E)** (Top) Percent of regions bound by any of the shown TFs, or by the three factors in conjunction with Esrrb or Nr5a2, showing changes in binding of the indicated factor two days after inducing the loss of Esrrb (-E), in the absence of Nr5a2 (-N) or two days after inducing the loss of Esrrb in the absence of Nr5a2 (-E-N). (Bottom) Boxplot, presented as in Fig S5B, showing the fold change in Oct4, Sox2 and Nanog binding at the same set of regions and in the same conditions. **(F)** Western blot showing the levels of Esrrb, Oct4, Sox2, Nanog and Lamin B1 in EKOiE ESCs or in EKOiE NrKO ESCs before (++, -N) or 2days after withdrawal of doxycycline (-E, and -E-N respectively). **(G)** Number of regions at which TF binding is affected or not by different combinations of depletions of Esrrb, Nr5a2 and Nanog (top). A grey rectangle (bottom) indicates regions responding to a given TF, white indicates independent regions. **(H)** Enrichment, over the intersection expected by chance (Fisher's exact test), of the regions affected by loss of the indicated TF pairs. **(I)** Boxplot, presented as in Fig S5B with single datapoints as dots, showing the fold change in Esrrb and Nr5a2 binding 2 days after the depletion of Esrrb (-E) and in the absence of Nr5a2 (-N), at a subset of the regions identified in figure 3B, which harbor only one Esrrb/Nr5a2 binding site. **(J)** Boxplot, presented as in Fig S5I, showing the fold change in Oct4 binding after depletion of Nr5a2 and/or Esrrb, at a subset of the regions identified in figure 3B, which harbor only one Esrrb/Nr5a2 binding site (-E,-N-,-E-N as in Fig S5E).

Development • Supplementary information

# *FULL SUPPLEMENTARY METHODS*

## Culture and Generation of ES cells.

### *General culture conditions.*

ES cells were cultured on 0.1% gelatine (Sigma-Aldrich, Cat# G1890-100G) in DMEM + GlutaMax-I (Gibco, Cat# 31966-021), 10% FCS (Gibco, Cat# 10270-098), 100 µM 2-mercaptoethanol (Gibco, Cat# 31350-010), 1× MEM non-essential amino acids (Gibco, Cat# 1140-035) and 10 ng×ml$^{-1}$ recombinant LIF (MILTENYI BIOTEC, Cat# 130-099-895) (Referred to as FCS/LIF). Cells were passaged 1:10 every 2–3 days. When indicated, cells were grown in 2i-containing medium (1µM PD0325901 and 3 µM CHIR99021; Axon Medchem Bv) - 0.5× DMEM/F12 (Gibco, Cat# 31331093), 0.5×Neurobasal (Gibco, Cat# 21103049), 0.5% N2 supplement 100× (Gibco, Cat# 17502048), 1% B27 supplement 50× (Gibco, Cat# 17504044), 10µg/mL Insulin (Sigma-Aldrich, Cat# I1882-100MG), 2 mM L-Glutamine (Invitrogen, Cat# 91139), 0.05% BSA (Sigma-Aldrich, Cat# A3311-10G), 100 µM 2-mercaptoethanol (Gibco, Cat# 31350-010), 10 ng/ml recombinant LIF (MILTENYI BIOTEC, Cat# 130-099-895) (Referred to as 2i/LIF). All ESC lines are routinely tested for mycoplasma contamination.

### *Derivation of Esrrb-T2a-GFP and Nr5a2-T2a-GFP ESCs.*

E14Tg2a were nucleofected - using an Amaxa Nucleofector II with a Mouse ES Cell Kit (LONZA, Cat# VPH-1001) and selecting programme A23 - with 3µg of a linearised targeting vector designed to insert a T2a linker – Fluorescent protein – IRES Blasticidin$^{R}$ cassette at the stop codon of *Esrrb* (ENSEMBL transcript Esrrb-206) or *Nr5a2* (ENSEMBL transcript Nr5a2-205) and 1µg of pU6_CBh-Cas9-T2A-mCherry (Addgene no. 64324) driving expression of the

Development • Supplementary information

gRNAs 5′- TGTGCTGGGCCATCACACCT -3′ for *Esrrb* or 5′- GCTTCCAGGGGTGGGACTT -3′ for *Nr5a2*. 48 hours later blasticidin selection was added (5µg×ml$^{-1}$ ) and after 2 weeks single colonies were picked, expanded, and correctly targeted cells identified by PCR on genomic DNA followed by sequencing.

***Derivation of Nr5a2-GFP + Esrrb-mCherry ESCs*.**

E14Tg2a were nucleofected (See 1b) with 3µg of a linearised targeting vector designed to insert a T2a or 5×Gly linker – Fluorescent protein – IRES Blasticidin$^{R}$ cassette at the stop codon of *Nr5a2* (ENSEMBL transcript Nr5a2-205) and 1µg of pU6_CBh-Cas9-T2A-mCherry (Addgene no. 64324) driving expression of the gRNA 5′- GCTTCCAGGGGTGGGACTT -3′. 48 hours later blasticidin selection was added (5µg×ml$^{-1}$) and after 2 weeks single colonies were picked, expanded, and correctly targeted cells identified by PCR on genomic DNA followed by sequencing. An identical procedure was followed to derive Nr5a2-GFP+Esrrb-mCherry ESCs from heterozygous Nr5a2-GFP cells that were further nucleofected with 3µg of a linearised targeting vector designed to insert a 5×Gly linker – mCherry – IRES Puromycin$^{R}$ cassette at the stop codon of *Esrrb and* 1µg of pU6_CBh-Cas9-T2A-mCherry driving expression of the gRNA 5′- TGTGCTGGGCCATCACACCT -3′.

***Derivation of FLAG-Nr5a2 ESCs.***

E14Tg2a were nucleofected (See 1b) with 3µg of a linearised targeting vector designed to insert a LoxP – Puromycin$^{R}$ – LoxP – 3xFLAG – Gly5 – cassette at the start codon of *Nr5a2* (ENSEMBL transcript Nr5a2-205) and 1µg of pU6_CBh-Cas9-T2A-mCherry plasmid driving expression of the gRNA 5′- CCACTTTGGGCAGCATGACA -3′. After 1 week, resistant cells were further nucleofected with 3µg of a plasmid driving expression of the Cre recombinase, and plated at clonal density in medium containing doxycycline.

Development • Supplementary information

2 weeks later, single colonies were picked, expanded, and correctly targeted cells, which had excised the Puromycin$^R$ cassette, identified by PCR on genomic DNA and by immunofluorescence with an anti-Flag mouse monoclonal antibody (M2 clone - Sigma-Aldrich Cat# F3165). Three heterozygous targeted clones (clones 2, 17 and 22) were selected for further experiments.

***Derivation of EKOiE HA-AID-Nr5a2 ESCs.***

EKOiE ESCs (Festuccia et al. 2016) were nucleofected (See 1b) with 3µg of a linearised targeting vector designed to insert a LoxP - Puromycin$^R$ - LoxP - HA - auxin inducible degron - Gly5 cassette at the start codon of *Nr5a2* (ENSEMBL transcript Nr5a2-205) and 1µg of pU6_CBh-Cas9-T2A-mCherry plasmid driving expression of the gRNA 5′- CCACTTTGGGCAGCATGACA -3′. 48 hours later doxycycline was temporarily withdrawn (to silence the TetO-3xFlag-Esrrb-IRES-Puromycin$^R$ transgene present in EKOiE ESCs) and selection was added. After 4 days resistant cells were further nucleofected with 3µg of a plasmid driving expression of the Cre recombinase, and plated at clonal density in medium containing doxycycline. 2 weeks later, single colonies were picked, expanded, and correctly targeted cells, which had excised the Puromycin$^R$ cassette, identified by PCR on genomic DNA and by immunofluorescence with anti-HA mouse monoclonal (clone 12CA5 - Roche Cat# 11583816001). One homozygous targeted clones (clone 8) was selected for further experiments.

***Derivation of EKOiE NrKO and EKOiE NrKO Repair ESCs.***

EKOiE ESCs (Festuccia et al. 2016) were nucleofected (See 1b) with 3µg of an equimolar pool of two pU6_CBh-Cas9-T2A-mCherry plasmids driving expression of the gRNAs 5′- CCTCACGTGCGAAAGCTGCA -3′ or 5′- GACACTTTATCGCCACACAC -3′ to induce double strand breaks into the 4$^{th}$

Development • Supplementary information

Exon of *Nr5a2* (Nr5a2-205). 48 hours later single mCherry positive cells were FACS sorted into individual wells of a 96-well plate and expanded. Correctly targeted cells were identified by PCR on genomic DNA and sequencing. Two clones bearing deletions on both alleles (clone 2; 53 bp deletion = mm10_Chr1:136,944,963-136,945,016 : deletion 1 in the scheme below -- clone 4; 52 bp deletion = mm10_Chr1:136,944,963-136,945,015 and 1761 bp deletion = mm10_Chr1:136,944,362-136,946,119 : deletions 1 and 2 below) in the exon encoding the first half of Nr5a2 DNA binding domain (exon 4 in transcript ENSEMBL Nr5a2-205) were selected for further experiments. RNA-seq analysis of expression from the disrupted alleles in EKOiE NrKO clone 4 ESCs shows as expected that only gapped reads align to the deleted region (see scheme below): transcript either bypass the entire targeted exon, or include the deletion. All of the transcript variants we detected introduce frame-shifts in the Nr5a2 open reading frame, and, if expressed, would encode proteins of minimal length (the longest is 74aa, vs 499aa for WT Nr5a2). In addition, qPCR analysis using primers targeting the deleted region of *Nr5a2* confirmed that no WT Nr5a2 transcripts are detected in our knockout clones (See Fig. S3A).

Clone 4 EKOiE NrKO ESCs were further nucleofected with 1µg of a pU6_CBh-Cas9-T2A-mCherry plasmid driving expression of the gRNA 5′-GAAAAGGCAAACCTTGCCAC -3′ specific to the disrupted *Nr5a2* allele (short

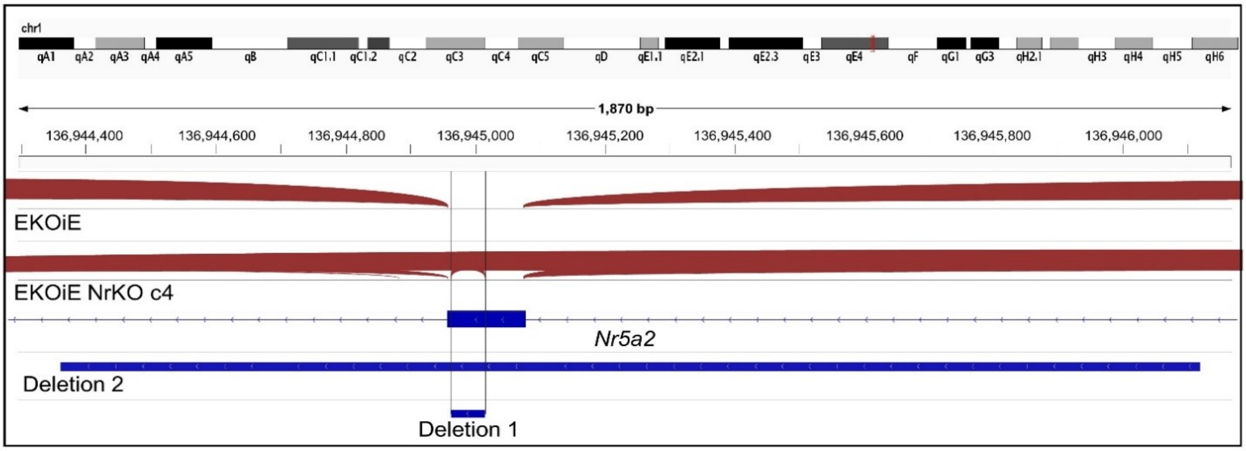

Span of the deletions occurred in EKOiE NrKO clones, along with a schematic representation of the splicing events in the transcript generated by WT and deleted alleles, as determined by RNA-seq. Transcripts either bypass entirely the exon including the Nr5a2 DNA binding domain, or include the truncated exon, leading to a frame shift in the coding sequence.

Development • Supplementary information

deletion), and 7 µg of a 300bp-long repair template obtained by PCR from E14Tg2a genomic DNA (primers 5′- TCAGCAATGCTTTTCAGTGCAG -3’ and 5’- TGACACTTCCCCCACCTCAC -3’). 48 hours later single mCherry positive cells were FACS sorted into individual wells of a 96-well plate and expanded. Correctly repaired cells were identified by PCR on genomic DNA and sequencing. qPCR analysis verified that expression of transcripts including the repaired exon was restored (See Fig. S3A). Two repaired clones (c4.2 and c16.1) were selected for further experiments.

Development • Supplementary information

## TF binding analysis by ChIP-seq.

### *Chromatin immunoprecipitation (ChIP).*

TF binding was assessed in E14Tg2a Flag-Nr5a2 ESCs cultured in FCS/LIF, and EKOiE, EKOiE NrKO, or EKOiE HA-AID-Nr5a2 ESCs cultured in 2i/LIF. Cells were trypsinised, counted and washed in PBS before replating ($2x10^6$ / 10cm dish) in the presence or absence of 1µg/ml doxycycline (Sigma-Aldrich, Cat# I5148) for 2 days. After trypsinisation, $10^7$ ES cells were crosslinked in 2 ml of freshly prepared PBS-DSG 2 mM at pH 7.0 (Sigma-Aldrich, Cat# 80424-5 mg) for 50 min at RT with occasional shaking. After pelleting and washing once in PBS, cells were incubated for 10 min in 2 ml PBS 1% formaldehyde (Thermo Scientific, Cat# 28908). Crosslinking was stopped with 0.125 mM glycine for 5 min at RT. Cells were pelleted and washed with ice-cold PBS. Cells were resuspended in 2 ml of swelling buffer (25 mM Hepes pH 7.95, 10 mM KCl, 10 mM EDTA) freshly supplemented with 1× protease inhibitor cocktail (PIC-Roche, Cat# 04 693 116 001) and 0.5% IGEPAL. After 30 min on ice, the suspension was passed 40 times in a dounce homogenizer. Cells were then centrifuged and resuspended in 300 µl of TSE150 (0.1% SDS, 1% Triton X-100, 2 mM EDTA, 20 mM Tris-HCl pH8, 150 mM NaCl) buffer, freshly supplemented with 1× PIC. Samples were sonicated in 1.5 ml tubes (Diagenode) using a Bioruptor Pico (Diagenode) for 7 cycles divided into 30 s ON–30 s OFF sub-cycles at maximum power, in circulating ice-cold water. After centrifugation (30 min, full speed, 4 °C), the supernatant was either used immediately for immunoprecipitation or stored at −80 °C until use, generally within the month. Five microliters were used to quantify the chromatin concentration and check DNA size (typically 200–350 bp). Chromatin from $10^7$ cells was used for each ChIP-seq after pre-clearing it for 3 hours rotating on-wheel at 4 °C in 300 µl of TSE150 containing 50 µl of protein G Sepharose beads (Sigma-Aldrich, Cat# P3296-5 ML) 50% slurry, previously blocked with BSA (500 µg×ml$^{-1}$; Roche, Cat# 5931665103) and

Development • Supplementary information

yeast tRNA (1 µg×ml$^{-1}$; Invitrogen, Cat# AM7119). Immunoprecipitations with anti-Esrrb mouse monoclonal (1µg per 2x10$^{6}$cells, Perseus Proteomics, Cat# H6-705-00), anti-Nanog rabbit polyclonal (0.6 µg per 2x10$^{6}$cells, Cosmobio, Cat# REC-RCAB001P); anti-Oct4 rabbit polyclonal (1 µg per 2x10$^{6}$cells, Abcam Cat # ab19857); anti-Sox2 rabbit polyclonal (1 µl per 2x10$^{6}$cells – concentration not specified, Active Motif Cat# 39844); anti-Flag mouse monoclonal (1 µg per 2x10$^{6}$cells Sigma-Aldrich Cat# F3165); anti-HA mouse monoclonal (1 µg per 2x10$^{6}$cells , clone 12CA5 - Roche Cat# 11 583 816 001) antibodies, were performed overnight rotating on-wheel at 4 °C in 500 µl of TSE150. 20 µl were set apart for input DNA extraction and precipitation. 25 µl of blocked protein G beads 50% slurry was added for 4 h rotating on-wheel at 4 °C. Beads were pelleted and washed for 5 min rotating on-wheel at RT with 1 ml of buffer in the following order: 3 × TSE150, 1 × TSE500 (as TSE150 but 500 mM NaCl), 1× washing buffer (10 mM Tris-HCl pH8, 0.25M LiCl, 0.5% NP-40, 0.5% Na-deoxycholate, 1 mM EDTA), and 2 × TE (10 mM Tris-HCl pH8, 1 mM EDTA). Elution was performed in 100 µl of elution buffer (1% SDS, 10 mM EDTA, 50 mM Tris-HCl pH 8) for 15 min at 65°C after vigorous vortexing. Eluates were collected after centrifugation and beads rinsed in 150 µl of TE-SDS1%. After centrifugation, the supernatant was pooled with the corresponding first eluate. For both immunoprecipitated and input chromatin, the crosslinking was reversed overnight at 65°C, followed by proteinase K treatment, phenol/chloroform extraction and ethanol precipitation.

***Library preparation.***

*SPRI Bead preparation:* 1 ml Sera-Mag™ Magnetic SpeedBeads™, carboxylated, 1 µm, 3 EDAC/PA5 (GE Healthcare Life Sciences, Cat# 65152105050250) were washed 3 times with a TE-Tween solution (10 mM Tris HCl pH 8, 1 mM EDTA, 0.05% Tween 20, pH 8.0) and resuspended in TE-

Development • Supplementary information

Tween-20% PEG 8000 solution (10 mM Tris HCl pH 8, 1 mM EDTA, 0.05% Tween 20, pH 8.0).

*End repair:* Precipitated DNA was resuspended in 37.5µl of water and mixed with 2µl of 10mM dNTPs, 5µl of NEB T4 ligase buffer, 2.5µl of NEB T4 polymerase (Cat# M0203L), 0.5µl of NEB Klenow polymerase (Cat# M0210L) and 2.5µl of NEB T4 PNK (Cat# M0201L). Samples were incubated 30min at 20ºC in a thermocycler. DNA was purified with SPRI beads: 90µl of SPRI bead suspension and 50µl isopropanol were added and samples transferred to a 96 well plate. After incubating for 5 min, the plate was put on a 96S Super Ring Magnet (Alpaqua, Cat# A001322), beads were allowed to separate completely, and the supernatant removed without disrupting the bead pellet. Beads were washed twice with 200µl of 70% Ethanol and the supernatant completely removed. DNA was eluted in 21µl of water.

*A-Tailing:* 20µl of sample were mixed with 2.5µl of NEB Buffer #2, 1µl of 5mM dATP, 1.5µl of NEB Klenow 3'-5' exo minus (Cat# M0212L), and incubated at 37ºC for 30min in a thermocycler. DNA was purified with SPRI beads as before, but using a volume of 45µl of beads and 25µl isopropanol. DNA was elute in 20µl of water.

*Adaptor ligation:* 19µl of sample were mixed with 2.5µl of NEB T4 ligase buffer, 1.25µl of a 0.2µM solution of annealed adapters, and 2.5µl of NEB concentrated T4 ligase (Cat# M0202M) and incubated overnight at 16ºC. DNA was purified with SPRI beads as before, but using a volume of 35µl of beads and no isopropanol, eluting in 20 µl of water. Adaptors were designed in house based on the structure of Illumina TruSeq™ indexed and forked adapters, modified to include extended indexes. 22.5µl each of 40µM ssDNA Barcoded and Universal adaptor solutions were mixed with 5µl of NEB buffer 2, and annealed in a thermocycler.

Development • Supplementary information

Barcoded adaptor:
5’P-GATCGGAAGAGCACACGTCTGAACTCCAGTCAC-Index -ATCTCGTATGCCGTCTTCTGCTTG.
5’P indicates the presence of a 5’phosphate group.

Universal adapter:
AATGATACGGCGACCACCGAGATCTACACTCTTTCCCTACACGACGCTCTTCCGATC*T.
* Indicates a phosphorothioate bond between the last C and T.

*Library amplification:* 19.5 µl of sample were mixed with 1µl of a 1:10 dilution of Quant-iT Picogreen dye (Invitrogen, Cat# P11496), 25µl of KAPA HiFi HotStart 2× master Mix (Kapa Bioscience Cat# KK2502), 1µl of 10µM PCR 1.0 and 1µl of PCR 2.0 primers (See below). The amplification mix was distributed in two wells of a LightCycler 384 plate (Roche, Cat# 4729749001) and on a LightCycler 480 II instrument (Roche, Cat# 05015243001) using the following program: 1’ at 98°C; N cycles: 10” at 98°C; 20” at 64°C; 45” at 72°C. The number of cycles N was determined on real time by monitoring the fluorescence such that the amplification was stopped during the exponential phase. Samples were removed from the plate and purified with 70 µl SPRI beads without isopropanol, and eluting in 40µl of water. 1µl was used to measure the DNA concentration with a Qubit 3 and the provided reagents (Invitrogen, Cat#Q33218). 1 ng of DNA was used to check fragment size with a D1000 High Sensitivity Screentape and appropriate reagents (Agilent, Cat# 5067-5584, Cat# 5067-5585) on an Agilent 2200 Tapestation.
*Library sequencing:* the libraries were sequenced (paired-end 150bp reads) by Novogene Co. Ltd.

Development • Supplementary information

***Gene expression analysis by RT-qPCR*.**

RNA was prepared with a Nucleospin RNA kit following the manufactured instructions, and performing DNAse I treatment (Macherey-Nagel, Cat# 740955.50). RNAs were resuspended in Ultrapure DNAse/RNase Free Distilled Water (Thermo Scientific, Cat# 10977035), and 1ug of RNA was used for cDNA preparation using a Transcriptor First Strand cDNA synthesis kit (Roche, Cat# 04897030001), using random hexamer priming, performing a denaturation step for 10 min at 65°C, and then incubating 10 min at 25°C, 30 min at 55°C and 5 min at 85°C. Real-time RT–PCR reactions were performed in triplicate in 384-wells plates with a 480 LightCycler (Roche) using a LightCycler 480 SYBR Green I Master mix (Roche cat. 04887352001). Five microliters of cDNA were used per reaction. Values for gene expression were normalised to the levels of TATAbox Binding Protein (TBP).  PCR primer sequences are listed below:

Development • Supplementary information

Pou5f1: Fw CCCCAATGCCGTGAAGTTG; Rv TCAGCAGCTTGGCAAACTGTT
Sox2: Fw CACAGATGCAACCGATGCA; Rv GGTGCCCTGCTGCGAGTA
Nanog: Fw AGGATGAAGTGCAAGCGGTG; Rv TGCTGAGCCCTTCTGAATCAG
Zfp42: Fw CAGCTCCTGCACACAGAAGA; Rv ACTGATCCGCAAACACCTG
Nr5a2: Fw CTCCTCACGTGCGAAAGCTG; Rv GGGACATCGTTTTCTCTGCGT
TBP: Fw GGGGAGCTGTGATGTGAAGT; Rv CCAGGAAATAATTCTGGCTCA

**Western Blot.**

For Western Blot analysis cell pellets corresponding to $10^6$ cells were resuspended in 100 µl RIPA Buffer (Tris/Cl pH 7.5 10mM, NaCl 150mM, EDTA 0.5mM, SDS 0.1%, Triton X-100 1%, Deoxycholate 1%) supplemented with 1× protease inhibitor cocktail (Roche, Cat# 04693116001) and incubated for 2h in the presence of 2500 U/ml benzonase (Sigma-Aldrich, Cat# E1014) at 4°C. After centrifugation (10 min, full speed, 4 °C), the supernatant was recovered and mixed with an equal volume of 2x Laemmli Sample Buffer (BIO-RAD, Cat# 161-0737) containing β-mercaptoethanol, boiled for 10 min at 95°C and centrifuged for 10 min at maximum speed. 20 µl per sample was loaded on 10% Mini-PROTEAN® TGX Stain-Free™ Protein Gels, 10 well, 30 µl (BIO-RAD, Cat# 4568033) and run in 1× SDS-Running Buffer (250 mM Tris/ 1.92 M Glycine/1% SDS) at 10-20 mA using the Mini-PROTEAN Tetra System (BIO-RAD). Proteins were transferred on a Protran nitrocellulose membrane (Amersham, Cat# 10600003) for 1 hour at 300mA using a wet transfer system (BIO-RAD) in 1× Transfer Buffer (10× 0.25M Tris/ 1.92M Glycine) prepared with a final concentration of 20% Ethanol. Membranes were blocked in PBSTw (PBS 0.1% Tween-20) 5% BSA for 1 hour at RT and incubated over night at 4°C with primary antibodies (diluted in PBSTw 5% BSA). Excess antibodies were washed with PBSTw (5 washes, 5 min each) and membranes incubated for 1 hour at RT in Alexa Fluor dye or HRP-conjugated secondary antibodies (diluted in PBSTw 5% BSA). Membranes were washed 5 times, 10 min each at RT and, for HRP antibodies, incubated

Development • Supplementary information

with PIERCE ECL2 Western Blotting Substrate (Thermo Scientific, Cat# 80196) 5 min in dark. After excess reagent was removed, proteins were visualised using a BIO-RAD Chemidoc MP Imaging System and processed using the Image Lab Software (BIO-RAD).
Primary antibodies used were: Rabbit polyclonal anti-Lamin B1 (Abcam, Cat# ab16048) (1:10000), goat polyclonal anti-Sox2 (R&D, Cat# AF2018) (1:1000), anti-Esrrb mouse monoclonal (Perseus Proteomics, Cat# H6-705-00) (1:1000), anti-Nanog rabbit polyclonal (Cosmo Bio, Cat# REC-RCAB001P) (1:1000); anti-Oct4 rabbit polyclonal (Abcam Cat # ab19857) (1:1000). Secondaries: anti-Rabbit IgG-HRP (Thermo Scientific , Cat# RB230254) (1:5.000), Alexa Fluor 488 AffiniPure Donkey Anti-Mouse IgG (H+L) (Jackson ImmunoResearch, Cat #715-545-150); Alexa Fluor 647 AffiniPure Donkey Anti-Goat IgG (H+L) (Jackson ImmunoResearch, Cat # 705-605-147).

## Computational Methods.

### *Data and availability.*

A total of 69 ChIP-seq and 20 RNA-seq libraries were generated and sequenced: ChIP-seq was performed in triplicates; FCS/L RNA-seq in duplicates; 2i/L RNA-seq in triplicates. All datasets are available in the GEO database under the accession number GSE152186.

### *ChIP-seq analyses.*

Paired end reads were trimmed by aligning read pairs to discover regions of reverse complementarity surrounded by adapters, alignment and trimming were performed with the packge SequenceTrimmer.jl (https://github.com/owensnick/SequenceTrimmer.jl) with Julia 0.6 (Bezanson et al., 2017). Reads were aligned with Bowtie2 (Langmead and Salzberg, 2012) to the mm10 genome, with options “-k 10”. Reads were additionally

Development • Supplementary information

filtered for those with a single discovered alignment (in Bowtie2 “k” mode this is mapping quality = 255) and an edit distance less than 4. Duplicate reads (those aligning with same left-right coordinate) were collapsed into one. Peaks were called against relevant inputs for all samples using MACS2 (Feng et al., 2012) with “callpeak -q 0.2 -g mm”. Peaks intersecting with the mm10 blacklist (Encode_Project_Consortium, 2012) were excluded. We further excluded an outlying replicate for Sox2 in 2i/LIF -E-N identified by principal components analysis and the fraction of reads in peaks of 5.3% as compared to 17.9% and 21.9% for the other two replicates. To determine a set of candidate binding regions bound by Nr5a2 or Esrrb in FCS/LIF we required that a peak must be called in all 3 replicates for either Esrrb or Nr5a2. To determine binding regions in 2i/LIF, for each TF we required that a peak must be called in all replicates of a given condition, with the exception of Nr5a2 for which a peak had to be called in one out of two replicates. We then merged the peaks of each TF analysed in FCS/L or 2i/L, respectively, to obtain regions were multiple TF bind. To quantify ChIP signal at each of these merged regions we took the mean signal over replicates from the original TF peak if present, and took the mean signal over the merged interval if a TF peak was not present. For the Venn diagram and the heatmap shown in Figure 1 E-F and S2D-F, regions bound by Esrrb and Nr5a2 in FCS/LIF or 2i/LIF were further merged, intersections with the original sets calculated, and read coverage profiles quantified over the merged regions. The heatmap was simplified by averaging groups of 80 regions. For k-means clustering of regions bound in 2i/LIF, as offered by the Clustering package of Julia (Bezanson et al., 2015), we selected regions in which a peak was called for Oct4, Sox2, Nanog and either Esrrb or Nr5a2 and normalized binding levels at each region for each factor to the condition displaying maximal binding. The heatmap in Figure 3B was simplified by averaging results of groups of 20 regions.  The Venn diagram in Figure S5A displays the overlap between regions called as bound by Esrrb or Nr5a2, or conjunctly by Oct4, Sox2 and Nanog in all replicates for each factor, in at least one condition (with the

Development • Supplementary information

exception of Nr5a2 for which regions were included if called as bound in one out of two replicates). To determine the number of clusters to be used we employed the proximity enrichment to differentially expressed genes (DEGs) described below, and found k=5 was the first k at which the presented ChIP region classes were visible and the enrichment of DEGs in proximity to ChIP-seq peaks robust. The clusters were further complemented by differential binding analysis using DESeq2 (Love et al., 2014). We set size factors according to total mapped library reads, and employed design ~ChipInput + Esrrb + Nr5a2 + Esrrb:Nr5a2 aimed at determining the effect of Esrrb and Nr5a2 depletion and their interaction over input. We tested for Esrrb loss, Nr5a2 loss and the loss of both against the presence of both TFs. Gene Ontology analyses of each cluster were made with GREAT using standard parameters. ChIP-seq data visualization for Figures 1D and 3A was made using the following R packages and assisted by ggplot2 (Wickham, 2016): ChIP-seq profiles were extracted from Bigwig files using rtracklayer (Lawrence et al., 2009), smoothed with zoo (Zeileis and Grothendieck, 2005) and plotted using the Gviz (Hahne, 2016) and GenomicFeatures (Lawrence et al., 2013) R packages. All enrichment heatmaps and metaplots were computed with bamsignals (Mammana, 2020); Venn diagrams were made with eulerr (Larsson, 2020), heatmaps were made with ComplexHeatmap (Gu et al., 2016b). De-novo motif discovery for Figure 1G was performed on the ensemble of Esrrb/Nr5a2 bound regions in FCS/L using the Regulatory Sequence Analysis Tools (RSAT) through their web-based interface with standard parameters (rsat.sb-roscoff.fr)(Nguyen et al., 2018). For figure 1H, and S2F, a matrix for the highest ranking discovered motif was exported, trimmed and plotted using the TFBSTools R package (Tan and Lenhard, 2016). Two PFM matrixes were created to reflect a perfect match to the consensus sequence TCA AGG **T**CA or TCA AGG **C**CA, and occurrence of these motifs in Esrrb and Nr5a2 bound regions was determined using TFBSTools. Only matches displaying 0 or 1 mismatch to the consensus, while requiring an exact match to either variant of the 7$^{th}$ base, were considered,

Development • Supplementary information

and the highest scoring matches to the motif was retained for each region. For Figures 1I, 3D and S2E, a motif corresponding to the Jaspar Nr5a2 motif MA0505.1 was trimmed to the consensus TCAAGG**X**CA, where **X** represents complete freedom at the 7$^{th}$ base. Motifs occurrences were identified and the frequency of T or C at the 7$^{th}$ base weighted by motif scores within each region determined using MotifScanner.jl (https://github.com/exeter-tfs/MotifScanner.jl). For Figure 3D, to correlate relative changes on Oct4 binding with changes in the frequency of C or T at the 7$^{th}$ base, we took the Zscore of fold changes of Oct4 occupancy in -E, -N, and -E-N compared to +E+N ordered by the C/T ratio. We looked for consistent changes in Zscored fold change with C/T ratio by Gaussian process (GP) regression using (GaussianProcesses.jl - https://github.com/STOR-i/GaussianProcesses.jl) with squared exponential kernel, and we ploted GP mean and GP function standard deviation confidence intervals. To further develop the relationship between motif occurrence and TF occupancy, we scanned the mm10 genome for occurrences of Jaspar motifs: Esrrb - MA0141.3, Nr5a2 MA0505.1 – trimmed as described above, and Oct4/Sox2 MA0142.1, using FIMO (Grant et al., 2011) with parameters '--thresh 1e-3 --max-stored-scores 50000000' and supplied a 0-order Markov background file describing the relative nucleotide frequencies in the mm10 assembly. We intersected motifs with all considered regulatory regions. In Figure S5C, to determine the dependence of Nr5a2 binding on Nr5a2 motifs, we performed a linear regression of log mean reads per 10 million per peak to the maximal motif score in both +E+N and -E conditions and present the $R^2$ scores. For Figure 3F, to calculate enrichments of Oct/Sox motifs at a given distance from Esrrb/Nr5a2 motifs we take all motifs with FIMO motif score greater than the median for that motif and calculate the absolute distance between all pairs of Esrrb and Oct4/Sox2 motif midpoints over all regions. We then calculate an empirical background distribution by calculating the motif mid-point distance between motifs placed at random with the interval of each peak region. We repeat

Development • Supplementary information

this 20,000 times per region and use results to calculate the expected number of motif pairs to be found at a given distance. This expectation is then used to derive a Poisson p-value for the observed number of pairs of motifs with mid-points at each distance. For Figure S3I-J, To determine regions with a single recognisable Esrrb/Nr5a2 motif, we selected those regions in which FIMO scans for Esrrb and Nr5a2 motifs identified the same single sequence and no other. As our FIMO parameters are permissive and detect many low scoring motif we are confident that we have excluded regions that contain a secondary weak Esrrb or Nr5a2 motif.

***RNA-seq analyses.***

Stranded paired end RNA-seq reads were aligned to the mm10 genome using STAR (Dobin et al. 2013) and quantified by RSEM (Li and Dewey, 2011) using the RSEM-STAR pipeline, with additional options "--seed 1618 --calc-pme --calc-ci --estimate-rspd --paired-end". RSEM estimated read counts per sample were rounded for use with DESeq2 (Love et al., 2014). Genes with at least 20 raw counts in all replicates of at least one condition were considered for differential expression analysis. For all differential expression tests DESeq2 was run without independent filtering; genes considered with absolute FC > 1.5 and FDR < 0.01 were considered as differentially expressed. Gene Ontology analyses (Table S3) were carried out in PANTHER (geneontology.org) using standard parameters. For families of related terms, only the most specific hierarchical level was retained. General data visualization was made in R using ggplot2 (Wickham, 2016) and ComplexHeatmaps packages (Gu et al., 2016b). In Figure 4C, to determine enrichments of each group of differentially expressed genes in proximity to the ChIP-seq clusters, we calculated Hypergeometic right tail p-values for for the association between differentially expressed genes within x bp of a ChIP-seq peak belonging to a cluster, compared to a background of all genes within x bp of a cluster peak,  for x in [1, 1e+6] bp, using the Julia package

Development • Supplementary information

ProximityEnrichment.jl (https://github.com/owensnick/ProximityEnrichment.jl).

***Comparisons to published datasets.***

For Figure S5G-H, comparison to Nanog dependent regions from (Heurtier et al., 2019) was performed by liftover of mm9 available regions to mm10, followed by the intersection of Nanog responsive loci (Heurtier Clusters 1-4), with Esrrb responsive (our Clusters 1, 2) and Nr5a2 responsive elements (our clusters 1,3). For Figure 4E, Oct4 ChIP-seq datasets from (Buecker et al., 2014) and (Festuccia et al., 2018) were obtained through the European Nucleotide Archive database and aligned with Bowtie2 (Langmead and Salzberg, 2012) to the mm10 genome, with default options. Output sam files were converted to bam, sorted, and indexed using Samtools (Li et al., 2009). Coverage in the set of Esrrb/Nr5a2 dependent or independent regulatory regions identified in this study was quantified in each external dataset using the R packages bamsignals (Mammana, 2020), Rsamtools (Morgan, 2020), and GenomicRanges (Lawrence et al., 2009), and data plotted with ggplot2 (Wickham, 2016). For Figure 4D, processed data for RNA-seq correlations were obtained from supplementary tables available in the following publications, and differentially genes selected according to the following criteria:

- (Dunn et al., 2019):
  Up in 2i/LIF vs PD/LIF (Activated by CH) – Fold change > 2, Absolute value in 2i/LIF> 5 FPKM (after averaging replicates)
  Down in 2i/LIFL vs PD/LIF (Repressed by CH) – Fold change < 0.5, Absolute value in PD/LIF > 5 FPKM (after averaging replicates)
- (Ye et al., 2013):
  Up in LIF+CH / LIF (Activated by CH) – Fold change >2, detection p-value <0.1
  Down in LIF+CH / LIF (Repressed by CH) – Fold change <0.5, detection p-value <0.1

Development • Supplementary information

- (Martello et al., 2013):
  Upregulated after LIF treatment (1h) in Stat3 wild-type cells cultured in 2i – p-value < 0.05
  Downregulated after LIF treatment (1h) in Stat3 wild-type cells cultured in 2i – p-value < 0.05
- (Yi et al., 2011):
  Upregulated in $Tcf3^{-/-}/Tcf3^{+/+}$ – Fold change <0.5 (after averaging replicates)
  Downregulated in $Tcf3^{-/-}/Tcf3^{+/+}$ – Fold change >1.5 (after averaging replicates)
- (Boroviak et al., 2015):
  Upregulated in ICM3.5/Epi5.5 – Fold change > 2, Absolute value > 5 (after averaging replicates)
  Downregulated in ICM3.5/Epi5.5 – Fold change < 0.5, Absolute value > 5 (after averaging replicates)
  Upregulated in Epi4.5/Epi5.5 – Fold change > 2, Absolute value > 5 (after averaging replicates)
  Downregulated in Epi4.5/Epi5.5 – Fold change < 0.5, Absolute value > 5 (after averaging replicates)
- (Buecker et al., 2014):
  Upregulated in ESC/EpiLC – Fold change > 2, Absolute value > 5 FPKM
  Downregulated in ESC/EpiLC – Fold change < 0.5, Absolute value > 5 FPKM
- (Festuccia et al., 2018):
  Upregulated in $Esrrb^{High}/Esrrb^{Negative}$ – Fold change > 2 (after averaging replicates)
  Downregulated in $Esrrb^{High}/Esrrb^{Negative}$ – Fold change < 0.5 (after averaging replicates)

In the case of (Argelaguet et al., 2019), tables of differential gene expression between the conditions presented in the figures were re-calculated using the scripts available at (https://github.com/rargelaguet/scnmt_gastrulation), which gave identical results to those reported by the authors, but allowed to include in our analyses a tailored sets of comparisons. Differentially expressed genes were selected according to the following criteria: Up in each of the conditions/ E4.5 epiblast – Fold change >1.5, detection p-value <0.01;

Development • Supplementary information

Down in each of the conditions / E4.5 epiblast – Fold change <(1/1.5), detection p-value <0.01. Pearson correlation coefficients were then calculated based on the fold changes in expression of each gene in the external datasets and the fold change in expression observed after depletion of Esrrb, Nr5a2 or both Esrrb and Nr5a2 in FCS/LIF or 2i/LIF. Heatmaps displaying the correlation between datasets were generated using the ComplexHeatmap R package (Gu et al., 2016b). For Figure S2A, processed data for RNA-seq analysis of Esrrb and Nr5a2 relative levels of expression was obtained from supplementary tables available in the following publications: (Atlasi et al., 2019; Buecker et al., 2014; Cruz-Molina et al., 2017; Dunn et al., 2019; Ficz et al., 2013; Gu et al., 2016a; Hsu et al., 2019; von Meyenn et al., 2016). For Figure S2B, processed single cell RNA-seq (Kolodziejczyk et al., 2015) was obtained from the ESpresso database (https://espresso.teichlab.sanger.ac.uk/), and data imported in and plotted with the FlowJo software suite.

### *ADDITIONAL REFERENCES*


- Argelaguet, R., Clark, S.J., Mohammed, H., Stapel, L.C., Krueger, C., Kapourani, C.A., Imaz-Rosshandler, I., Lohoff, T., Xiang, Y., Hanna, C.W., *et al.* (2019). Multi-omics profiling of mouse gastrulation at single-cell resolution. Nature *576*, 487-491.
- Atlasi, Y., Megchelenbrink, W., Peng, T., Habibi, E., Joshi, O., Wang, S.Y., Wang, C., Logie, C., Poser, I., Marks, H., *et al.* (2019). Epigenetic modulation of a hardwired 3D chromatin landscape in two naive states of pluripotency. Nat Cell Biol *21*, 568-578.
- Bezanson, J., Edelman, A., Karpinski, S., and Shah, V. (2015). Julia: A fresh approach to numerical computing. https://arxivorg/abs/14111607.
- Boroviak, T., Loos, R., Lombard, P., Okahara, J., Behr, R., Sasaki, E., Nichols, J., Smith, A., and Bertone, P. (2015). Lineage-Specific Profiling Delineates the Emergence and Progression of Naive Pluripotency in Mammalian Embryogenesis. Dev Cell *35*, 366-382.
- Buecker, C., Srinivasan, R., Wu, Z., Calo, E., Acampora, D., Faial, T., Simeone, A., Tan, M., Swigut, T., and Wysocka, J. (2014). Reorganization of enhancer patterns in transition from naive to primed pluripotency. Cell Stem Cell *14*, 838-853.


Development • Supplementary information

- Cruz-Molina, S., Respuela, P., Tebartz, C., Kolovos, P., Nikolic, M., Fueyo, R., van Ijcken, W.F.J., Grosveld, F., Frommolt, P., Bazzi, H., *et al.* (2017). PRC2 Facilitates the Regulatory Topology Required for Poised Enhancer Function during Pluripotent Stem Cell Differentiation. Cell Stem Cell *20*, 689-705 e689.
- Dunn, S.J., Li, M.A., Carbognin, E., Smith, A., and Martello, G. (2019). A common molecular logic determines embryonic stem cell self-renewal and reprogramming. EMBO J *38*.
- Encode_Project_Consortium (2012). An integrated encyclopedia of DNA elements in the human genome. Nature *489*, 57-74.
- Feng, J., Liu, T., Qin, B., Zhang, Y., and Liu, X.S. (2012). Identifying ChIP-seq enrichment using MACS. Nat Protoc *7*, 1728-1740.
- Festuccia, N., Halbritter, F., Corsinotti, A., Gagliardi, A., Colby, D., Tomlinson, S.R., and Chambers, I. (2018). Esrrb extinction triggers dismantling of naive pluripotency and marks commitment to differentiation. EMBO J *37*.
- Ficz, G., Hore, T.A., Santos, F., Lee, H.J., Dean, W., Arand, J., Krueger, F., Oxley, D., Paul, Y.L., Walter, J., *et al.* (2013). FGF signaling inhibition in ESCs drives rapid genome-wide demethylation to the epigenetic ground state of pluripotency. Cell Stem Cell *13*, 351-359.
- Grant, C.E., Bailey, T.L., and Noble, W.S. (2011). FIMO: scanning for occurrences of a given motif. Bioinformatics *27*, 1017-1018.
- Gu, K.L., Zhang, Q., Yan, Y., Li, T.T., Duan, F.F., Hao, J., Wang, X.W., Shi, M., Wu, D.R., Guo, W.T., *et al.* (2016a). Pluripotency-associated miR-290/302 family of microRNAs promote the dismantling of naive pluripotency. Cell Res *26*, 350-366.
- Gu, Z., Eils, R., and Schlesner, M. (2016b). Complex heatmaps reveal patterns and correlations in multidimensional genomic data. Bioinformatics *32*, 2847-2849.
- Hahne, F. (2016). Visualizing Genomic Data Using Gviz and Bioconductor, in Statistical Genomics. Methods and Protocols. Anticancer Res *36*, 3224.
- Heurtier, V., Owens, N., Gonzalez, I., Mueller, F., Proux, C., Mornico, D., Clerc, P., Dubois, A., and Navarro, P. (2019). The molecular logic of Nanog-induced self-renewal in mouse embryonic stem cells. Nat Commun *10*, 1109.
- Hsu, J., Arand, J., Chaikovsky, A., Mooney, N.A., Demeter, J., Brison, C.M., Oliverio, R., Vogel, H., Rubin, S.M., Jackson, P.K., *et al.* (2019). E2F4 regulates transcriptional activation in mouse embryonic stem cells independently of the RB family. Nature Communications *10*.
- Kolodziejczyk, A.A., Kim, J.K., Tsang, J.C., Ilicic, T., Henriksson, J., Natarajan, K.N., Tuck, A.C., Gao, X., Buhler, M., Liu, P., *et al.* (2015). Single

Cell RNA-Sequencing of Pluripotent States Unlocks Modular Transcriptional Variation. Cell Stem Cell *17*, 471-485.

- Langmead, B., and Salzberg, S.L. (2012). Fast gapped-read alignment with Bowtie 2. Nat Methods *9*, 357-359.
- Larsson, J. (2020). eulerr: Area-Proportional Euler and Venn Diagrams with Ellipses. R package version 6.1.0.
- Lawrence, M., Gentleman, R., and Carey, V. (2009). rtracklayer: an R package for interfacing with genome browsers. Bioinformatics *25*, 1841-1842.
- Lawrence, M., Huber, W., Pages, H., Aboyoun, P., Carlson, M., Gentleman, R., Morgan, M.T., and Carey, V.J. (2013). Software for computing and annotating genomic ranges. PLoS Comput Biol *9*, e1003118.
- Li, B., and Dewey, C.N. (2011). RSEM: accurate transcript quantification from RNA-Seq data with or without a reference genome. BMC Bioinformatics *12*, 323.
- Li, H., Handsaker, B., Wysoker, A., Fennell, T., Ruan, J., Homer, N., Marth, G., Abecasis, G., Durbin, R., and Genome Project Data Processing, S. (2009). The Sequence Alignment/Map format and SAMtools. Bioinformatics *25*, 2078-2079.
- Love, M.I., Huber, W., and Anders, S. (2014). Moderated estimation of fold change and dispersion for RNA-seq data with DESeq2. Genome Biol *15*, 550.
- Mammana, A. (2020). bamsignals: Extract read count signals from bam files. R package version 1.20.0.
- Martello, G., Bertone, P., and Smith, A. (2013). Identification of the missing pluripotency mediator downstream of leukaemia inhibitory factor. EMBO J *32*, 2561-2574.
- Morgan, M. (2020). Rsamtools: Binary alignment (BAM), FASTA, variant call (BCF), and tabix file import. R package version 2.4.0.
- Nguyen, N.T.T., Contreras-Moreira, B., Castro-Mondragon, J.A., Santana-Garcia, W., Ossio, R., Robles-Espinoza, C.D., Bahin, M., Collombet, S., Vincens, P., Thieffry, D., *et al.* (2018). RSAT 2018: regulatory sequence analysis tools 20th anniversary. Nucleic Acids Res *46*, W209-W214.
- Tan, G., and Lenhard, B. (2016). TFBSTools: an R/bioconductor package for transcription factor binding site analysis. Bioinformatics *32*, 1555-1556.
- von Meyenn, F., Iurlaro, M., Habibi, E., Liu, N.Q., Salehzadeh-Yazdi, A., Santos, F., Petrini, E., Milagre, I., Yu, M., Xie, Z., *et al.* (2016). Impairment of DNA Methylation Maintenance Is the Main Cause of Global Demethylation in Naive Embryonic Stem Cells. Mol Cell *62*, 983.

- Wickham, H. (2016). ggplot2: Elegant Graphics for Data Analysis. Springer-Verlag New York.
- Ye, S., Li, P., Tong, C., and Ying, Q.L. (2013). Embryonic stem cell self-renewal pathways converge on the transcription factor Tfcp2l1. EMBO J *32*, 2548-2560.
- Yi, F., Pereira, L., Hoffman, J.A., Shy, B.R., Yuen, C.M., Liu, D.R., and Merrill, B.J. (2011). Opposing effects of Tcf3 and Tcf1 control Wnt stimulation of embryonic stem cell self-renewal. Nat Cell Biol *13*, 762-770.
- Zeileis, A., and Grothendieck, G. (2005). zoo: S3 infrastructure for regular and irregular time series. J Stat Softw *14*.


Development • Supplementary information

Table S1. Summary of ChIP-seq results in FCS/LIF

Click here to download Table S1

Table S2. Gene expression analysis

Click here to download Table S2

Table S3. Gene ontology analysis on deferentially expressed genes

Click here to download Table S3

Table S4. Summary of ChIP-seq results in 2i/LIF

Click here to download Table S4
