# Supplementary material for: The combined action of Esrrb and Nr5a2 is essential for murine naïve pluripotency
Source: Development. 2021 Sep 10;148(17):dev199604. doi: 10.1242/dev.199604 (PMC8451941; doi:10.1242/dev.199604)
Supplement: Reviewer comments [file develop-148-199604_review_history.pdf]

The combined action of Esrrb and Nr5a2 is essential for murine naive pluripotency

Nicola Festuccia, Nick Owens, Almira Chervova, Agnès Dubois and Pablo Navarro
DOI: 10.1242/dev.199604

Editor: Maria Elena Torres-Padilla

Review timeline
Original submission: 14 March 2021
Editorial decision: 14 April 2021
First revision received: 23 July 2021
Accepted: 4 August 2021

Original submission

First decision letter

MS ID#: DEVELOP/2021/199604

MS TITLE: The combined action of Esrrb and Nr5a2 is essential for naive pluripotency

AUTHORS: Nicola Festuccia, Nick Owens, Almira Chervova, Agnès Dubois, and Pablo Navarro-Gil

I have now received all the referees' reports on the above manuscript, and have reached a decision. The referees' comments are appended below, or you can access them online: please go to BenchPress and click on the 'Manuscripts with Decisions' queue in the Author Area.

As you will see, the referees express interest in your work, but are split in their opinions and have raised some criticisms and recommend a revision of your manuscript before we can consider publication. If you are able to revise the manuscript along the lines suggested, which may involve further experiments, I will be happy receive a revised version of the manuscript. In particular, it would be important to tidy up the discussion and portray clearer the contribution of your work as a demonstration of functional redundancy between these two orphan NRs. I would also strongly encourage you to attend all the suggestions on additional analyses by Reviewer 3 as well as clarify, either through experiments or further analysis and/or discussion, the concern on Reviewer 2 (also shared by Reviewer 3) on your conclusion that OSN binding is controlled by Esrrb and Nr5a2.

Your revised paper will be re-reviewed by one or more of the original referees, and acceptance of your manuscript will depend on your addressing satisfactorily the reviewers' major concerns. Please also note that Development will normally permit only one round of major revision.

We are aware that you may be experiencing disruption to the normal running of your lab that make experimental revisions challenging. If it would be helpful, we encourage you to contact us to discuss your revision in greater detail. Please send us a point-by-point response indicating where you are able to address concerns raised (either experimentally or by changes to the text) and where you will not be able to do so within the normal timeframe of a revision. We will then provide further guidance. Please also note that we are happy to extend revision timeframes as necessary.

Please attend to all of the reviewers' comments and ensure that you clearly highlight all changes made in the revised manuscript. Please avoid using 'Tracked changes' in Word files as these are lost in PDF conversion. I should be grateful if you would also provide a point-by-point response detailing

© 2021. Published by The Company of Biologists under the terms of the Creative Commons Attribution License (https://creativecommons.org/licenses/by/4.0/).

how you have dealt with the points raised by the reviewers in the 'Response to Reviewers' box. If you do not agree with any of their criticisms or suggestions please explain clearly why this is so.

Reviewer 1

Advance summary and potential significance to field

In this manuscript, the authors show that the joint inactivation of two orphan nuclear receptors – Esrrb and Nr5a2 – causes mouse ESCs to exit pluripotency even when the cells are cultured in stringent 2i/Lif conditions. This is an interesting result because only a small number of transcription factors have similar strong effects when disrupted in 2i-naive cells. The phenotype is certainly very striking. This study therefore reinforces Esrrb and Nr5a2 as central and potentially direct regulators of Oct4, Sox2 and Nanog function and activators of shared target genes, and in doing so advances our understanding of the gene regulatory control of naïve pluripotency. However, the significance of the main finding is tempered by previous studies that identified orphan nuclear receptors, including Esrrb and Nr5a2, as having prominent roles in reprogramming to naïve pluripotency, co-binding with Oct4, Sox2 and Nanog, promoting pluripotent factor expression such as Oct4 and Nanog, and early developmental phenotypes (e.g. PMIDs: 20096661, 20734354, 22977234; 24341592; 29910149). It is interesting to see the phenotype following the inactivation of both Esrrb and Nr5a2, although it is perhaps not all that unexpected for two prominent pluripotency-associated orphan nuclear receptors to have redundant roles in maintaining ESCs. Additionally, the authors propose that Esrrb and Nr5a2 function cooperatively but, as detailed below, there is insufficient evidence to support this conclusion, and it is unclear to what extent the two factors have independent versus redundant roles. The broader significance of the work is therefore less clear.

Comments for the author

Comments on the main conclusions of the manuscript:
1. “Conjunt activity of Esrrb and Nr5a2 are strictly required for the self-renewal of ESCs and pluripotency in naïve mESCs.” The results show convincingly that the inactivation of Esrrb and Nr5a2 causes loss of self-renewal and pluripotency exit. But the current results do not provide sufficient support that Esrrb and Nr5a2 are required cooperatively, or whether this represents independent roles or functional redundancy between two factors. More direct analysis of cooperative function would be required to support this conclusion, which is an important aspect of the manuscript.

2. “Esrrb and Nr5a2 co-occupy regulatory elements”. This is well supported by the data, although this result has also been reported previously in other studies (e.g. PMIDs 18555785; 20096661; 29910149).

3. “Esrrb and Nr5a2 control the binding of Oct4, Sox2 and Nanog to DNA.” The authors show that Oct4, Sox2 and Nanog ChIP-seq signals are reduced at target sites when Esrrb and Nr5a2 are both inactivated. It is not clear though if this is a direct or indirect effect, or how this might be happening, and therefore the conclusion that Esrrb and Nr5a2 “control the binding of Oct4, Sox2 and Nanog to DNA” is not fully supported by the data.

4. “In the absence of Esrrb and Nr5a2, the pluripotency network collapses and the cells differentiate.” This conclusion is well supported by the data.

Additional specific comments:
1. In Figures 1H and 1I, the correspondence between ChIP signal and the different motif categories is fairly modest, and does not support the strong claim that Esrrb and Nr5a2 show “exquisitely specific, DNA binding preferences”. Further evidence would be needed here, potentially through gel shift assays, for instance, or even editing target motifs, although there might be other ways too. Also, I think that the crystal structure of the Nr5a2 DNA binding domain has been published – could that information contain any additional supporting evidence for why ‘C’ might be preferred to ‘T’? However, it is also important to add that the authors don’t really return to this finding until the discussion, and it is difficult to tell whether motif preferences could have a role in Esrrb and

© 2021. Published by The Company of Biologists under the terms of the Creative Commons Attribution License (https://creativecommons.org/licenses/by/4.0/).

Nr5a2 function. If the authors have additional data then that is ideal, but instead of starting new experiments, it might be better to simply (but substantially) tone down this result and the conclusions drawn from it.

2. Figures 2A and B. The authors "…conclude that Esrrb and Nr5a2 cooperate to support pluripotency in FCS/LIF" with "clear additive effects". I don't see this from Figure 2B – it looks as though in the absence of Esrrb, the cells behave in a very similar way irrespective of whether Nr5a2 is present or not. The lack of gene expression changes when Nr5a2 is deleted suggests that Esrrb is operating normally in the absence of Nr5a2, and it is difficult to see evidence for cooperativity. Similarly, the cells seem fine when Esrrb is removed and Nr5a2 is in place. In contrast, the data using 2i/Lif cells presented in Figures 2E and 2F are more convincing.

3. Does Esrrb or Nr5a2 binding change when the other factor is factor is deleted? This might help to get at redundant versus independent functions.

4. I could not see any evidence that Nr5a2 protein is removed in the targeted lines? Are there any suitable antibodies to provide this evidence? If not, can the authors be sure that the protein is gone and/or there is no truncated protein, for instance?

Minor comments
1. Please indicate the species in the title.
2. Second paragraph of the introduction. Consider breaking up the long sentence to help readability.
3. Figure S2B – please indicate what the units are on the axes.
4. The findings reported in Figures 2C and G could be strengthened by adding "-E +N repair" data.
5. The same images are shown in Figure 2H and Figure S3D. I don't think this is necessary.
6. Presumably "harms" should be "arms"?
7. Discussion: "Nr5a2 ablation results in gastrulation defects, and a severe phenotype emerges only at E7.5".
But that seems to be at odds with what is reported in the Gu et al paper where the authors conclude that "…
LRH-1-/- [Nr5a2-/-] embryos die around E6.5."
8. Many of the figures lack any statistical analysis – please add.
9. Please add scale bars to images, and molecular weight markers to blots.

Reviewer 2

Advance summary and potential significance to field

The mechanisms that sustain pluripotency of mouse ESCs have been extensively studied over the last decade and a consensus has emerged of the core transcriptional network that maintains this state. However, gaps remain, in particular about the basis for the underlying cause of the robustness of the pluripotency network in 2i conditions. Here the authors address this question focusing on previous observations on the expression and function of Esrrb whose deletion has been shown to have strong effects for self renewal in FCS conditions but not in 2i. The authors show that, in 2i conditions, the loss of Esrrb is functionally compensated by the activity of Nr5a2, another member of he same family of transcription factors, and that the deletion of both leads to a collapse of the activity of the network i.e they act redundantly in 2i. The authors do a very thorough job in demonstrating this functional redundancy, the underlying molecular basis and mechanism. This study goes a long way towards closing an important gap in our understanding of mouse pluripotency in vitro.

Comments for the author

The report is technically excellent and I do not see the need for any additional experiments. However, it would be good if they could tidy up the discussion wich I found a bit too long and, in places, repetitive. Also and importantly, the all important relationship between signalling and their observations is confusing and, in places, rather than trying to shoehorn speculation, they should

© 2021. Published by The Company of Biologists under the terms of the Creative Commons Attribution License (https://creativecommons.org/licenses/by/4.0/).

state that they don't know. In this context, the authors may want to consider the findings of Zhang et al (PMID 23505158) on the role of Wnt signalling in 2i conditions.

Reviewer 3

Advance summary and potential significance to field

In this manuscript Festuccia et al. describe the redundancy between two nuclear receptors Esrrb and Nr5a2. They generate an elegant series of reporters, mutants and repaired alleles in mouse embryonic stem cells (ESCs), characterize the binding of both factors, the loss of function phenotypes, and the binding of pluripotency transcription factors (TFs) Oct4, Sox2, Nanog in the different mutant lines. They then assess the extent to which alterations in pluripotency TF binding correlate with the transcriptional state of mutant cells, and how these states relate to the transition between naÃ¯ve and primed pluripotency.

These findings are of clear interest to the scientific community, and we would like to compliment the authors on a well written and conducted study of the potential redundancy between the 2 orphan nuclear receptors, Esrrb and Nr5a2, in naÃ¯ve pluripotency. We recommend this manuscript for publication in DEVELOPMENT, provided the authors address the points below. Our comments primarily concern the interpretation of the dataset and strengthening the correlations included.

Comments for the author

General comments:
1. As there are specific effects attributable to both nuclear receptors, what is the argument of redundancy between the two factors, apart from the family relationship? What makes the double mutant phenotype described here unique to the combination of these two factors? As the network is indeed highly interconnected and interdependent, one could imagine that the double KO of other auxiliary pluripotency TFs would also cause the pluripotency network wiring to collapse. This could obviously be addressed with additional mutants, but we understand this would represent a significant amount of additional work.
We suggest the authors provide analysis of whether there is specific movement of Esrrb to Nr5a2 sites in the absence of Nr5a2 and vice versa. In addition, we would like the authors to carry out more detailed motif analysis of the identified E+N peaks, to determine whether one motif is present and competed for, or two motifs are present side by side. In the instance that there are two motifs, their spatial relationship to each other could be significant. The authors also speculate about heterodimeric binding – could that be supported by motif analysis? Perhaps they could also discuss this idea in the wake of structural and biochemical data on the binding of these factors to DNA. The above suggestions are in line with the authors discussion at the end of page 12, and we find further analysis in this direction, would greatly strengthen the paper.

2. We realize that in murine ESCs, pluripotency and self-renewal are intertwined properties that are hard to disentangle. We wonder whether the observed phenotypes in the clonal assays result from a collapse of pluripotency, or a proliferation/self-renewal defect.
To this end we wonder whether the double KO cells (as the two single KO’s) can be propagated and passaged in bulk culture (is density sufficient to preserve stemness)? We find it would strengthen the paper to include an experiment where the cells are passaged and counted over ~10 days/ 5 passages, as +E+N, -E+N, +E-N and -E-N, complimented by some analysis of the identity of these cells.

3. We realize, that ~50% of the transcripts in both the FSC/LIF and the 2i/LIF RNA-seq (fig. 1F and 4A), consist of activated genes upon DB KO. What are these many genes and what are their GO-annotations? In general, it would improve the manuscript and strengthen the message of the paper to include the complete GO-annotation lists for the major RNA-seq clusters and ChIP-seq clusters identified, as supplementary figures or tables.

4. The authors state that Esrrb and Nr5a2 control the binding of Oc4, Sox2 and Nanog to DNA. Yet, to say that the factors control the binding of OSN, implies they are above them in the hierarchy. Is it in fact so that Esrrb or Nr5a2 stay bound in the absence of the other pluripotency factors? Or is it

© 2021. Published by The Company of Biologists under the terms of the Creative Commons Attribution License (https://creativecommons.org/licenses/by/4.0/).

likely that the stability of pluripotency TF binding on DNA is an emergent property of the network, where all factors have equal importance?

5. The authors mention cooperativity as a mechanism for stable DNA binding of pluripotency TFs. However, if this is the case, it would be interesting to look for the OSN motif in the Esrrb/ Nr5a2 peaks identified. This would shed light into how Esrrb/Nr5a2 acts to recruit OSN.

6. The authors perform extensive and careful analysis of their own data, but we find the comparison to other datasets limited and therefor hard to interpret. The correlation analysis in figure 4D focuses on naïve vs primed pluripotency and WNT/ERK signaling. We suggest they include more defined states, such as rosette stage pluripotency, extraembryonic mesoderm and endoderm, and post gastrulation ectoderm, mesoderm and endoderm. It would strengthen the interpretation greatly to show, that the loss of Esrrb and Nr5a2 triggers a conversion specifically towards primed pluripotency, and not just a general loss of pluripotency and undirected spontaneous differentiation.

7. The authors speculate about a role of Nr5a2 and Esrrb specifically in naïve pluripotency and state that the downregulation of Esrrb and Nr5a2 likely is crucial to trigger the conversion to EpiLCs/primed pluripotency. Yet the loss of Esrrb and Nr5a2 results in silencing of the key EpiSC/primed pluripotency TF Otx2, among others. As the authors point out, this is seemingly contradictory. Perhaps the authors could differentiate ESC to EpiLC after 2 days of Esrrb and Nr5a2 withdrawal in 2iL – will the cells be less, more or similarly competent to convert? They could also extend the discussion of this ‘dual role’ of Esrrb and Nr5a2 in Epi specification.

Specific comments:
1: Figure 1F could nicely be supplemented by a Venn diagram of the overlap of Esrrb and Nr5a2 binding sites.

2: Please include the top 10 hits for all GO-analysis conducted in supplementary. For example, page 6:
”Since genes activated and repressed by both factors are enriched in terms such as response to LIF (FDR=2.27e-24) and terms linked to differentiation (e.g. morphogenesis; FDR=2.32e-57), respectively, we conclude that Esrrb and Nr5a2 cooperate to support pluripotency in FCS/LIF.”
Another example is on the end of page 8.

3: On page 6, the following two sections seem to be a repetition:
“Gene expression analysis further supports this conclusion, since acute Esrrb depletion in 2i/LIF results in reduced expression of both auxiliary and core pluripotency genes, in particular Klf4, Tbx3, Tfcp2l1 and Sox2, and in the mild upregulation of differentiation markers (Fig. 2G, S3C).”
“In line, gene expression analysis revealed the collapse of pluripotency gene expression and the upregulation of a panel of differentiation markers just two days after acute loss of Esrrb and Nr5a2 function (Fig. 2G, S3C).”
4: Figure 4D was tricky to grasp initially. Please add to the y-axis or figure legend that it is the ratio ++/KO

First revision

Author response to reviewers' comments

Reviewer 1 Advance Summary and Potential Significance to Field:
In this manuscript, the authors show that the joint inactivation of two orphan nuclear receptors - Esrrb and Nr5a2 - causes mouse ESCs to exit pluripotency even when the cells are cultured in stringent 2i/Lif conditions. This is an interesting result because only a small number of transcription factors have similar, strong effects when disrupted in 2i-naive cells. The phenotype is certainly very striking. This study therefore reinforces Esrrb and Nr5a2 as central and potentially direct regulators of Oct4, Sox2 and Nanog function and activators of shared target genes, and in doing so advances

© 2021. Published by The Company of Biologists under the terms of the Creative Commons Attribution License (https://creativecommons.org/licenses/by/4.0/).

our understanding of the gene regulatory control of naïve pluripotency. However, the significance of the main finding is tempered by previous studies that identified orphan nuclear receptors, including Esrrb and Nr5a2, as having prominent roles in reprogramming to naïve pluripotency, co-binding with Oct4, Sox2 and Nanog, promoting pluripotent factor expression such as Oct4 and Nanog, and early developmental phenotypes (e.g. PMIDs: 20096661, 20734354, 22977234; 24341592; 29910149). It is interesting to see the phenotype following the inactivation of both Esrrb and Nr5a2, although it is perhaps not all that unexpected for two prominent pluripotency-associated orphan nuclear receptors to have redundant roles in maintaining ESCs. Additionally, the authors propose that Esrrb and Nr5a2 function cooperatively but, as detailed below, there is insufficient evidence to support this conclusion, and it is unclear to what extent the two factors have independent versus redundant roles. The broader significance of the work is therefore less clear.

We are pleased that the reviewer finds that the phenotype we report is “striking”, recognises that few pluripotency TFs are known to be strictly required for the maintenance of pluripotency in ground state culture conditions (2i/LIF), and that therefore our study “advances our understanding of the gene regulatory control of naïve pluripotency”. However, we disagree with the reviewer’s opinion that the finding that Esrrb and Nr5a2 have redundant role in supporting pluripotency is not unexpected.

In fact, **the double knockout phenotype we present is not the predictable consequence of previous reports analysing individual mutants, rather it is in contrast and puts in context earlier studies**. That Esrrb KO compromises the self-renewal of ESCs grown in differentiation-permissive conditions (FCS+LIF) is undisputable, as we published in Cell Stem Cell (Festuccia et al., Cell Stem Cell, 2012). However, the group of A Smith showed that the loss of Esrrb in ground-state conditions (2i+LIF) is inconsequential (Martello et al., Cell Stem Cell, 2012). Later, the group of H Stunnenberg refined the initial conclusions from Festuccia et al. and Martello et al., showing only a transient detrimental effect for the inactivation of Esrrb, evident in cells challenged with a shift in culture conditions (Atlasi et al, Nature Cell Biology 2019). Yet, none of the studies published to date justifies concluding that Esrrb is essential for the self-renewal of ESC. Interestingly, the same paper reports that the deletion of Nr5a2 is quasi inconsequential in ES cells, in line with previous studies (Fujii, Scientific Reports, 2015, Gu et al., Mol Cell Biol, 2005). **Therefore, based on current knowledge, the concomitant loss of Esrrb and Nr5a2 should be well tolerated in ground-state culture conditions.**

Reviewer 1 Comments for the Author:

Comments on the main conclusions of the manuscript:

1. “Conjunt activity of Esrrb and Nr5a2 are strictly required for the self-renewal of ESCs and pluripotency in naïve mESCs.” The results show convincingly that the inactivation of Esrrb and Nr5a2 causes loss of self-renewal and pluripotency exit. But the current results do not provide sufficient support that Esrrb and Nr5a2 are required cooperatively, or whether this represents independent roles or functional redundancy between two factors. More direct analysis of cooperative function would be required to support this conclusion, which is an important aspect of the manuscript.

Several lines of evidence support our claims that Esrrb and Nr5a2 perform redundant and closely related roles in supporting pluripotency: 1/ The two orphan receptors are highly related in terms of structure, share the general mechanism of recruitment of coactivators characteristic of nuclear receptors, and display a common mode of DNA binding. In agreement, 2/ we clearly show and extensive overlap in the DNA binding profile of the two factors, which 3/ is justified by the ability of the two TFs to bind similar motifs on DNA. Moreover, 4/ we show that the gene expression changes triggered by the loss of Esrrb and Nr5a2 are highly related, and provide evidence of a concordant and additive effect on many pluripotency and developmental genes.

Despite these considerations, we agree that this is an original and important aspect of the manuscript. To respond to the reviewer’s concern, and to other points raised by reviewer 3 (see points 4, 5 and general remarks), we now provide additional evidence in support of the notion that Esrrb and Nr5a2 act cooperatively. First, we show that the changes in TF binding triggered in response to the inactivation of Esrrb and Nr5a2 are much more mutually related than either is to the response to the acute depletion of an unrelated pluripotency factor, Nanog (Fig. S5G, H). Second, we identify regions targeted by Esrrb and Nr5a2 that include only one biding site for these

© 2021. Published by The Company of Biologists under the terms of the Creative Commons Attribution License (https://creativecommons.org/licenses/by/4.0/).

TFs, and show that: 1/ Esrrb and Nr5a2 reciprocally facilitate each other's access to these common sites (binding cooperativity), and 2/ by acting at the very same positions on DNA, Esrrb and Nr5a2 additively affect the occupancy by other regulators (functional cooperativity – Fig. S5I,J). While it is natural to expect that other pluripotency TFs are co-occupying some of the loci targeted by Esrrb and Nr5a2, the respective binding sites will be broadly distributed over these regulatory regions. This leads to a variety of possible functional interactions dependent on the grammar of motifs at these loci, and on effects mediated by the chromatin and recruited cofactors. Esrrb and Nr5a2 act instead at a common set of motifs, and exert a clearly concordant action.

2. "Esrrb and Nr5a2 co-occupy regulatory elements". This is well supported by the data, although this result has also been reported previously in other studies (e.g. PMIDs 18555785; 20096661; 29910149).

While it is true that the laboratory of HH Ng has mapped before the genomewide binding profile of Nr5a2, occupancy was only broadly compared to that of several other pluripotency TFs, and not of Esrrb in specific. Also the group of H Stunnenberg (Atlasi et al, Nature Cell Biology 2019) has performed ChIP-seq for Nr5a2, but binding was assessed in ESCs during the switch from FCS/LIF to 2i/LIF. Our study fill existing gaps and improves on previous reports by 1/ analysing binding of Nr5a2 protein expressed from its endogenous locus, and therefore at physiological levels; 2/ rigorously comparing binding of Esrrb and Nr5a2 in both FCS/LIF and 2i/LIF. This analysis revealed that the overlap in DNA binding between Esrrb and Nr5a2 is largely independent of the rewiring of the pluripotency network observed during the switch between these two culture conditions, and that the number or genomic regions targeted by Nr5a2 increases substantially in 2i/LIF, providing a basis to understand the importance of this TFs in the ground state.

3. "Esrrb and Nr5a2 control the binding of Oct4, Sox2 and Nanog to DNA." The authors show that Oct4, Sox2 and Nanog ChIP-seq signals are reduced at target sites when Esrrb and Nr5a2 are both inactivated. It is not clear though if this is a direct or indirect effect, or how this might be happening, and therefore the conclusion that Esrrb and Nr5a2 "control the binding of Oct4, Sox2 and Nanog to DNA" is not fully supported by the data.

We point out that we make no absolute claims as to which mechanisms might be driving the observed dependence in binding, and temper our interpretations by stating ("the reduced levels of Nanog and Sox2 protein 2 days after Nr5a2 and Esrrb depletion may partially contribute to these effects"). Yet, several consideration are in support of a direct effect. 1/ Our analysis was performed only 2 days after the acute depletion of Esrrb in Nr5a2 knockout lines, which includes the time required for Esrrb expression to drop following doxycycline withdrawal. 2/ Oct4 protein levels do not change in this timeframe, and Sox2 and Nanog are only mildly reduced (Fig. S5F), making it unlikely for the effects observed to be entirely secondary to changes in expression of other pluripotency regulators. 3/ As discussed in the text, the effect observed at Esrrb/Nr5a2 bound regions is significantly stronger than the reduction of binding observed at the ensemble of sites of pluripotency TF binding irrespective of Esrrb occupancy (Fig. S5D). 4/ We now provide evidence that the magnitude of the effects triggered by the loss of either Esrrb or Nr5a2 at regulatory regions is correlated to the presence of a binding motif variant preferred by one or the other nuclear receptor (Fig. 3D). Finally, 5/ we show that at regions that strongly depend on Esrrb and Nr5a2, Oct4/Sox2 composite motifs are weaker and closer to those for Esrrb/Nr5a2, compared to independent regions (Fig. 3E-F). The two last observations supports the notion that the direct recruitment, and the local activity on the chromatin by Esrrb and Nr5a2, are responsible for the effects we report. Overall, we agree that a complete characterisation of the mechanisms driving the dependence in binding between Esrrb/Nr5a2 and other TFs is key, but this will be the subject of future studies.

4. "In the absence of Esrrb and Nr5a2, the pluripotency network collapses and the cells differentiate." This conclusion is well supported by the data.
We are pleased that the reviewer finds that our claim that Esrrb and Nr5a2 are determinant in ensuring the function of the pluripotency gene regulatory network is well supported by the data.

Additional specific comments:
In Figures 1H and 1I, the correspondence between ChIP signal and the different motif categories is fairly modest, and does not support the strong claim that Esrrb and Nr5a2 show "exquisitely

© 2021. Published by The Company of Biologists under the terms of the Creative Commons Attribution License (https://creativecommons.org/licenses/by/4.0/).

specific, DNA binding preferences". Further evidence would be needed here, potentially through gel shift assays, for instance, or even editing target motifs, although there might be other ways too. Also, I think that the crystal structure of the Nr5a2 DNA binding domain has been published – could that information contain any additional supporting evidence for why 'C' might be preferred to 'T'? However, it is also important to add that the authors don't really return to this finding until the discussion, and it is difficult to tell whether motif preferences could have a role in Esrrb and Nr5a2 function. If the authors have additional data then that is ideal, but instead of starting new experiments, it might be better to simply (but substantially) tone down this result and the conclusions drawn from it.

We agree with the reviewer that the binding preference of Esrrb and Nr5a2 to the two variant of the motif is not absolute, and believe that our manuscript reflects this conclusion. Discussing our results we claim that "the specificity in binding to one or the other version of the motif is nuanced".

This finding is in agreement with published biochemical data. The 7th base of the motif (T/C) does not mediate strong interaction with estrogen related receptors or Nr5a2 in footprinting experiments (Yang et al., J Biol Chem 1996, Galarneau et al., Mol and Cell Biol 1996). In agreement, EMSA assays show that Nr5a2 can bind to motifs containing both T or C at the 7th position (Chen et al. J of Lipid Res. 2001, Hinshelwood et al. Mol Cell Endocrinol 2003, Clyne et al. 2002; JBC 277, 23:20591-20597, among others). As pointed out by the reviewer, and discussed in our manuscript, crystal structures are available for both Esrrb and Nr5a2 DNA binding domains (Gearhart et al. , 2003, J Mol Biol 327, 819-832; Solomon et al., 2005 J Mol Biol 354, 1091-1102; Weikum et al., 2016 J Mol Biol 428, 4981-4992). Importantly, the structure of Esrrb shows binding to the DNA sequence TCAAGG**T**CA, while that of Nr5a2 to TCAAGG**C**CA. Discussing the hNR5A2 structure the authors highlight that E104 (conserved in mouse) is responsible for establishing specific contacts with the 7th base pair of the motif (**C**:G): this residue is conserved in Esrrb. Similarly, in hESRRB, K128 (conserved in mouse) contacts the 7th base pair (**T**:A): as before, this residue is in conserved in Nr5a2. Thus, the binding preference of Esrrb and Nr5a2 does not result from differences in the residues contacting the discriminating base of the consensus binding site, and possibly is due to differences in the global organization of the DNA binding domain. We now discuss more in detail how these findings, and the known mode of Esrrb and Nr5a2 binding to DNA, might relate to our results.

In addition, to strengthen the evidence in support of a motif preference, we scanned through all the regions identified in our study, and show a continuous correlation between the prevalence of either motif at regulatory regions to the strength of Esrrb and Nr5a2 binding (Fig 1I). The reviewer also asks for evidence that the reported motif preference impacts Esrrb and Nr5a2 function. To address this point, we have shown there is a correlation between the presence of either motif at regulatory regions and the extent by which binding by other TFs depends on Esrrb and Nr5a2 (Fig 3D).

2. Figures 2A and B. The authors "…conclude that Esrrb and Nr5a2 cooperate to support pluripotency in FCS/LIF" with "clear additive effects". I don't see this from Figure 2B – it looks as though in the absence of Esrrb, the cells behave in a very similar way irrespective of whether Nr5a2 is present or not. The lack of gene expression changes when Nr5a2 is deleted suggests that Esrrb is operating normally in the absence of Nr5a2, and it is difficult to see evidence for cooperativity. Similarly, the cells seem fine when Esrrb is removed and Nr5a2 is in place. In contrast, the data using 2i/Lif cells presented in Figures 2E and 2F are more convincing.

As we have shown before, the phenotype of Esrrb deletion is drastic in clonal assays performed in FCS, as clearly shown in Fig. 2B, and supported by the gene expression changes in Fig. 2C. Also, an effect of Nr5a2 knockout on the efficiency of ESC self-renewal is evident, both in the presence (almost 3 fold reduction in undifferentiated colonies) and in the absence of Esrrb (eliminating the few colonies observed and resulting in the virtual absence of undifferentiated cells). Yet, we agree with the reviewer that Nr5a2 plays a minor role in FCS/LIF, as clearly stated in the manuscript ("This suggests that in FCS/LIF Esrrb plays a preponderant role that is nevertheless further supported by Nr5a2"). We believe this is due to the low expression levels of the TF in these conditions. To address the reviewer's concern we omitted the statement "with clear additive effects".

© 2021. Published by The Company of Biologists under the terms of the Creative Commons Attribution License (https://creativecommons.org/licenses/by/4.0/).

As the reviewer correctly points out, the phenotype of Nr5a2 deletion in 2i/LIF is more pronounced.

3. Does Esrrb or Nr5a2 binding change when the other factor is deleted? This might help to get at redundant versus independent functions.
We observe a reduction in Nr5a2 binding when Esrrb is deleted, and a milder reciprocal effect. While this data was already present in Fig. 3B-C, we added direct analyses showing that Esrrb and Nr5a2 cooperate to access individual binding sites on DNA (Fig. S5B, C, I).

4. I could not see any evidence that Nr5a2 protein is removed in the targeted lines? Are there any suitable antibodies to provide this evidence? If not, can the authors be sure that the protein is gone and/or there is no truncated protein, for instance?

Unfortunately, the production of the only antibody reported to reliably detect endogenous Nr5a2 by western blot in ESC (Heng et al. Cell Stem Cell, 2010) was discontinued. None of the other antibodies we tested (Abcam Cat# ab223211; Abcam Cat# ab189876; Abcam Cat# ab153944; EMD Millipore, Cat# MABE215) gave interpretable results.

However, a detailed characterisation of the genomic deletions present in our lines is reported in the method section of our manuscript. This confirmed that the sequence encoding the first zinc finger domain of Nr5a2 is absent in both knockout lines (two short deletions are present in one clone, and one short and one long deletion in the second. All deletions are depicted in the scheme

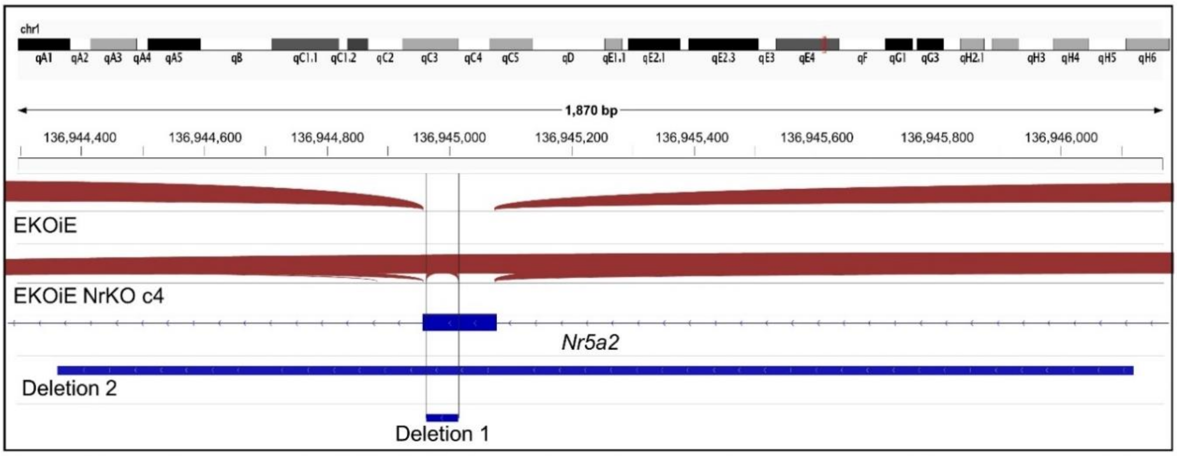

Span of the deletions occurred in EKOiE NrKO clones, along with a schematic representation of the splicing events in the transcript generated by WT and deleted alleles, as determined by RNA-seq. Transcripts either bypass entirely the exon including the Nr5a2 DNA binding domain, or include the truncated exon, leading to a frame shift in the coding sequence.

below). Moreover, qPCR analysis using primers targeting the deleted region of Nr5a2 indicate that no WT Nr5a2 transcript is detected in knockout ESCs. We further provide analysis of the residual transcripts originating from the deleted alleles, in the clone used for RNA-sequencing experiments (c4; one short, one long deletion). Transcripts either bypass entirely the exon encoding theNr5a2 DNA binding domain, or include truncated exons. All transcript variants we could detect carry frame-shifts in the Nr5a2 open reading frame, and, if expressed, would encode proteins of minimal length (the longest is 74aa long, vs 499aa for WT Nr5a2).

Minor comments

1. Please indicate the species in the title.

The title has been changed to “The combined action of Esrrb and Nr5a2 is essential for murine naïve pluripotency”.

2. Second paragraph of the introduction. Consider breaking up the long sentence to help readability.

The text has been changed accordingly.

© 2021. Published by The Company of Biologists under the terms of the Creative Commons Attribution License (https://creativecommons.org/licenses/by/4.0/).

3. Figure S2B – please indicate what the units are on the axes.

The panel has been changed accordingly.

4. The findings reported in Figures 2C and G could be strengthened by adding "-E +N repair" data.
We now provide analysis by q-PCR that indicate that the acute downregulation of pluripotency markers (Sox2 and Zfp42, as examples of general and naïve pluripotency TFs) observed after doxycyline withdrawal in EkoiE Nr5a2 KO ESCs, is rescued in repair clones (Fig S3E).

5. The same images are shown in Figure 2H and Figure S3D. I don't think this is necessary.

The repeated panels have been omitted.

6. Presumably "harms" should be "arms"?

The text has been changed accordingly.

7. Discussion: "Nr5a2 ablation results in gastrulation defects, and a severe phenotype emerges only at E7.5". But that seems to be at odds with what is reported in the Gu et al paper where the authors conclude that "… LRH-1−/− [Nr5a2-/-] embryos die around E6.5."

We understand the reviewer's concern. Three studies analysed the effect of Nr5a2 knockout during early development (Gu et al. Mol Cell Biol, 2005; Paré et al. JBC 2004; Labelle-Dumais et al., Dev Dyn, 2006), showing that substantial defects are observed around E6.5. The phenotype becomes even more severe at E7.5. Dying mutant embryos are nonetheless recovered up to E9.5. To better reflect this gradual worsening of the phenotype, the text will be amended to "Nr5a2 ablation results in gastrulation defects, and a severe phenotype emerges between E6.5 and E7.5"

8. Many of the figures lack any statistical analysis – please add.

The significance (FDR) of the gene expression changes observed in RNA-seq experiments is now highlighted in the figures, and tests of statistical significance have been added to panels presenting results of clonal plating (Fig. 2B,F), gene expression analysis by q-PCR (Fig. S3E, S4B), or growth curves (Fig. S4A). For other genomewide analyses, we stress that statistical measures applied to detect differences between distributions that include a high number of data points, as in the case for instance of our boxplots comparing TF binding at thousands of genomic regions, invariably lead to extreme values of significance. Such measures of statistical significance are therefore not informative, yet the statistical effects are (as seen in the difference between the boxplots).

9. Please add scale bars to images, and molecular weight markers to blots.

The panels have been changed accordingly.

Reviewer 2 Advance Summary and Potential Significance to Field:
The mechanisms that sustain pluripotency of mouse ESCs have been extensively studied over the last decade and a consensus has emerged of the core transcriptional network that maintains this state. However, gaps remain, in particular about the basis for the underlying cause of the robustness of the pluripotency network in 2i conditions. Here the authors address this question focusing on previous observations on the expression and function of Esrrb whose deletion has been shown to have strong effects for self renewal in FCS conditions but not in 2i. The authors show that, in 2i conditions, the loss of Esrrb is functionally compensated by the activity of Nr5a2, another member of the same family of transcription factors, and that the deletion of both leads to a collapse of the activity of the network ie they act redundantly in 2i. The authors do a very thorough job in demonstrating this functional redundancy, the underlying molecular basis and mechanism. This study goes a long way towards closing an important gap in our understanding of mouse pluripotency in vitro.

We are pleased that the reviewers finds our study rigorous and appreciates the importance of our findings.

© 2021. Published by The Company of Biologists under the terms of the Creative Commons Attribution License (https://creativecommons.org/licenses/by/4.0/).

Reviewer 2 Comments for the Author:
The report is technically excellent and I do not see the need for any additional experiments. However, it would be good if they could tidy up the discussion wich I found a bit too long and, in places, repetitive. Also and importantly, the all important relationship between signalling and their observations is confusing and, in places, rather than trying to shoehorn speculation, they should state that they don't know. In this context, the authors may want to consider the findings of Zhang et al (PMID 23505158) on the role of Wnt signalling in 2i conditions.

The discussion has been modified according to the reviewer's suggestions, in particular shortening the part regarding the relationship between the activity of Esrrb and Nr5a2, and that of developmental signaling pathways.

Reviewer 3 Advance Summary and Potential Significance to Field:

In this manuscript Festuccia et al. describe the redundancy between two nuclear receptors Esrrb and Nr5a2. They generate an elegant series of reporters, mutants and repaired alleles in mouse embryonic stem cells (ESCs), characterize the binding of both factors, the loss of function phenotypes, and the binding of pluripotency transcription factors (TFs) Oct4, Sox2, Nanog in the different mutant lines. They then assess the extent to which alterations in pluripotency TF binding correlate with the transcriptional state of mutant cells, and how these states relate to the transition between naïve and primed pluripotency.

These findings are of clear interest to the scientific community, and we would like to compliment the authors on a well written and conducted study of the potential redundancy between the 2 orphan nuclear receptors, Esrrb and Nr5a2, in naïve pluripotency. We recommend this manuscript for publication in DEVELOPMENT, provided the authors address the points below. Our comments primarily concern the interpretation of the dataset and strengthening the correlations included.

We thank the reviewer for the supportive comments and for pointing out that the "findings are of clear interest to the scientific community".

Reviewer 3 Comments for the Author:

General comments:

1. As there are specific effects attributable to both nuclear receptors, what is the argument of redundancy between the two factors, apart from the family relationship? What makes the double mutant phenotype described here unique to the combination of these two factors? As the network is indeed highly interconnected and interdependent, one could imagine that the double KO of other auxiliary pluripotency TFs would also cause the pluripotency network wiring to collapse. This could obviously be addressed with additional mutants, but we understand this would represent a significant amount of additional work.

We suggest the authors provide analysis of whether there is specific movement of Esrrb to Nr5a2 sites in the absence of Nr5a2 and vice versa. In addition, we would like the authors to carry out more detailed motif analysis of the identified E+N peaks, to determine whether one motif is present and competed for, or two motifs are present side by side. In the instance that there are two motifs, their spatial relationship to each other could be significant. The authors also speculate about heterodimeric binding - could that be supported by motif analysis? Perhaps they could also discuss this idea in the wake of structural and biochemical data on the binding of these factors to DNA. The above suggestions are in line with the authors discussion at the end of page 12, and we find further analysis in this direction, would greatly strengthen the paper.

In support of a unique functional overlap, we now show that the changes in TF binding triggered in response to the inactivation of Esrrb and Nr5a2 are more mutually related than either is to the response to the acute depletion of Nanog (Fig S5G, H).

Nonetheless, we agree that understanding how Esrrb and Nr5a2 functionally interact at common targets is key, and provide new evidence in support of a direct cooperativity between the two TFs. To this end, we have identified regions bound by both Esrrb and Nr5a2 that harbor only one

© 2021. Published by The Company of Biologists under the terms of the Creative Commons Attribution License (https://creativecommons.org/licenses/by/4.0/).

recognizable biding site, and show that: 1/ Esrrb and Nr5a2 reciprocally facilitate each other's access to these common targets (binding cooperativity– Fig. S5I), and 2/ by acting at the very same positions on DNA, Esrrb and Nr5a2 additively affect the occupancy by other regulators (functional cooperativity – Fig. S5J). While it is natural to expect that other pluripotency regulators are co-occupying some of the loci targeted by Esrrb and Nr5a2, these TFs act at sites that are broadly distributed over these regulatory regions. Their interactions are therefore indirect, leading to a wide array of possible functional outcomes. Esrrb and Nr5a2 molecules instead access the same sites on DNA, and in this sense engage in interactions that are not dissimilar to those between different molecules of the same type of TF. Yet, Esrrb and Nr5a2 could exert opposing activities at these targets. We exclude this possibility, and show a clear concordance in action, which may be consequent to a similar mechanism of recruitment of coactivators, characteristic of nuclear receptors. These new results provide evidence of a direct cooperativity, which is distinct from the broad synergy between unrelated pluripotency TF.

We have also extended the discussion of how published biochemical and structural data regarding the mode of Esrrb and Nr5a2 binding to DNA relate to our finding (See the response to reviewer 1, additional specific comments on page 4).

2. We realize that in murine ESCs, pluripotency and self-renewal are intertwined properties that are hard to disentangle. We wonder whether the observed phenotypes in the clonal assays result from a collapse of pluripotency, or a proliferation/self-renewal defect.
To this end we wonder whether the double KO cells (as the two single KO's) can be propagated and passaged in bulk culture (is density sufficient to preserve stemness)? We find it would strengthen the paper to include an experiment where the cells are passaged and counted over ~10 days/ 5 passages, as +E+N, -E+N, +E-N and -E-N, complimented by some analysis of the identity of these cells.

We have performed the analysis suggested by the reviewer. We show that passaging at high density is not sufficient to prevent the differentiation of ESCs in the absence of Esrrb and Nr5a2. Growth curves also indicate that the proliferation defects observed in double knockout ESCs become evident only following the loss of expression of pluripotency genes, and the appearance of clear signs of morphological differentiation. While cannot exclude a direct role for Esrrb and Nr5a2 in supporting proliferation, the notion that differentiated cells exit the cell cycle precludes establishing direct connections.

3. We realize, that ~50% of the transcripts in both the FSC/LIF and the 2i/LIF RNA-seq (fig. 1F and 4A), consist of activated genes upon DB KO. What are these many genes and what are their GO-annotations? In general, it would improve the manuscript and strengthen the message of the paper to include the complete GO-annotation lists for the major RNA-seq clusters and ChIP-seq clusters identified, as supplementary figures or tables.

While a brief discussion of the functional annotation of Esrrb and Nr5a2 target genes was already present in our manuscript, we now provide a complete lists of the gene ontology terms enriched in different clusters of differentially expressed genes (Supplementary table 3).

4. The authors state that Esrrb and Nr5a2 control the binding of Oc4, Sox2 and Nanog to DNA. Yet, to say that the factors control the binding of OSN, implies they are above them in the hierarchy. Is it in fact so that Esrrb or Nr5a2 stay bound in the absence of the other pluripotency factors? Or is it likely that the stability of pluripotency TF binding on DNA is an emergent property of the network, where all factors have equal importance?

Our results show that Esrrb and Nr5a2 control binding of other TFs at regulatory regions, and that the effect of the depletion of these TFs is major. Nonetheless, we agree that mutual dependence for access to DNA is a general feature of gene regulatory networks. For instance, we have previously shown that Nanog depletion affects Esrrb binding (Heurtier et al., Nat Com, 2019), and similar evidence is available for Oct4 (King and Klose, Elife, 2017). Yet the deletion of Nanog does not result in the collapse of the pluripotency network, and the loss of self-renewal. In this sense, our results establish a functional hierarchy, and lead to the proposal that Esrrb and Nr5a2 importance in supporting pluripotency parallels that of Oct4/Sox2.

© 2021. Published by The Company of Biologists under the terms of the Creative Commons Attribution License (https://creativecommons.org/licenses/by/4.0/).

The authors mention cooperativity as a mechanism for stable DNA binding of pluripotency TFs. However, if this is the case, it would be interesting to look for the OSN motif in the Esrrb/ Nr5a2 peaks identified. This would shed light into how Esrrb/Nr5a2 acts to recruit OSN.

We agree that identifying a potential mechanisms for the observed dependencies in DNA binding is important. Thus, we have explored the distribution of binding motifs for Oct4/Sox2 in the different classes of enhancers identified in our study. We show that, at enhancers that are strongly dependent on Esrrb and Nr5a2, Oct4/Sox2 binding sites are more pronouncedly enriched in proximity of those for the two orphan receptors, compared to independent regions. Moreover, at these loci Oct4/Sox2 sites tend to be more degenerate (Fig. 3 E,F). Thus, our analysis suggest that at dependent enhancers Oct4/Sox2 rely on local interactions with Esrrb and Nr5a2 - likely indirect and mediated by the chromatin - for accessing DNA.

6. The authors perform extensive and careful analysis of their own data, but we find the comparison to other datasets limited and therefor hard to interpret. The correlation analysis in figure 4D focuses on naïve vs primed pluripotency and WNT/ERK signaling. We suggest they include more defined states, such as rosette stage pluripotency, extraembryonic mesoderm and endoderm, and post gastrulation ectoderm, mesoderm and endoderm. It would strengthen the interpretation greatly to show, that the loss of Esrrb and Nr5a2 triggers a conversion specifically towards primed pluripotency, and not just a general loss of pluripotency and undirected spontaneous differentiation.

While we highlighted that a global activation of a transcriptional profile characteristic of primed pluripotency is induced after loss of Esrrb and Nr5a2, other signatures emerge in our datasets, as shown in Figure S3D. To better understand whether Esrrb/Nr5a2 suppress or promote differentiation into specific lineages, we now relate more extensively the transcriptional changes observed after depletion of the two factors to the expression profiles of different embryonic lineages (Fig 4D). Our analyses confirm the correlation between the gene expression changes observed after deletion of Esrrb and Nr5a2 and those characteristic of the transition from pre-to post-implantation in the epiblast. Nonetheless, such correlation is not exclusive, and a similarity to mesoderm, endoderm and ectoderm is also evident. It appears that, after exiting naïve pluripotency and possibly transiting through a formative/primed state, knockout ESCs undergo multilineage differentiation. This is now stated in the manuscript.

7. The authors speculate about a role of Nr5a2 and Esrrb specifically in naïve pluripotency and state that the downregulation of Esrrb and Nr5a2 likely is crucial to trigger the conversion to EpiLCs/primed pluripotency. Yet the loss of Esrrb and Nr5a2 results in silencing of the key EpiSC/primed pluripotency TF Otx2, among others. As the authors point out, this is seemingly contradictory. Perhaps the authors could differentiate ESC to EpiLC after 2 days of Esrrb and Nr5a2 withdrawal in 2iL – will the cells be less, more or similarly competent to convert? They could also extend the discussion of this 'dual role' of Esrrb and Nr5a2 in Epi specification.

While we agree that the ability of Esrrb to induce expression of key prime pluripotency TFs is intriguing, we stress that a similar phenotype was already described by the group of G Martello after the simple depletion of Esrrb (Carbognin et al., Cell Stem Cell Sneak Peek, 2020). Here we give new functional significance to these results reporting a strong additive effect of the additional loss of Nr5a2, which triggers the complete loss of Otx2 expression. The experiments suggested by the reviewer are indeed key. Yet, we feel they reach beyond the scope of our current manuscript, and deserve to be addressed thoroughly in a separate study. Also, to our knowledge, the work from Carbognin et al. has not yet passed peer review, and we are reluctant to include in our report work that significantly extends the conclusion of their study before publication.

Specific comments:

1: Figure 1F could nicely be supplemented by a Venn diagram of the overlap of Esrrb and Nr5a2 binding sites.

The diagram was included as supplementary material (Fig. S2D).

© 2021. Published by The Company of Biologists under the terms of the Creative Commons Attribution License (https://creativecommons.org/licenses/by/4.0/).

2: Please include the top 10 hits for all GO-analysis conducted in supplementary. For example, page 6:"Since genes activated and repressed by both factors are enriched in terms such as response to LIF (FDR=2.27e-24) and terms linked to differentiation (e.g. morphogenesis; FDR=2.32e-57), respectively, we conclude that Esrrb and Nr5a2 cooperate to support pluripotency in FCS/LIF." Another example is on the end of page 8.

The GO annotation of Esrrb and Nr5a2 responsive genes are now included as supplementary tables (Table S3).

3: On page 6, the following two sections seem to be a repetition:
"Gene expression analysis further supports this conclusion, since acute Esrrb depletion in 2i/LIF results in reduced expression of both auxiliary and core pluripotency genes, in particular Klf4, Tbx3, Tfcp2l1 and Sox2, and in the mild upregulation of differentiation markers (Fig. 2G, S3C)."
"In line, gene expression analysis revealed the collapse of pluripotency gene expression and the upregulation of a panel of differentiation markers just two days after acute loss of Esrrb and Nr5a2 function (Fig. 2G, S3C)."

While the first sentence refers to the effects of the acute deletion of Esrrb, the second describes the effects of the inactivation of both Esrrb and Nr5a2. To avoid confusion the first sentence has been changed to "Gene expression analysis further supports this conclusion, since acute the depletion of Esrrb **alone** in 2i/LIF results in reduced expression of both auxiliary and core pluripotency genes, in particular Klf4, Tbx3, Tfcp2l1 and Sox2, and in the mild upregulation of differentiation markers"

4: Figure 4D was tricky to grasp initially. Please add to the y-axis or figure legend that it is the ratio ++/KO

We now show in the figure the mention "correlation between the expression changes in –E and/or –N and:".

Second decision letter

MS ID#: DEVELOP/2021/199604

MS TITLE: The combined action of Esrrb and Nr5a2 is essential for naive pluripotency

AUTHORS: Nicola Festuccia, Nick Owens, Almira Chervova, Agnès Dubois, and Pablo Navarro-Gil
ARTICLE TYPE: Research Article

I am happy to tell you that your manuscript has been accepted for publication in Development, pending our standard ethics checks.

Reviewer 1

Advance summary and potential significance to field

The authors have done a good job in responding to my comments. In particular, the additional, more direct evidence to support cooperative functions between Esrrb and Nr5a2, and also the interplay with Oct4, Sox2 and Nanog, have strengthened the conclusions of the paper.

Comments for the author

-

Reviewer 2

© 2021. Published by The Company of Biologists under the terms of the Creative Commons Attribution License (https://creativecommons.org/licenses/by/4.0/).

Advance summary and potential significance to field

This has not changed from my last report

Comments for the author

No further revisions are needed. The authors have responded to the reviewers comments by addressing the criticisms and improving the manuscript.

Reviewer 3

Advance summary and potential significance to field

This is an important paper on the redundancy between the 2 orphan nuclear receptors, Esrrb and Nr5a2, in naÃ¯ve pluripotency.

Comments for the author

The authors revisions have improved the manuscript and it should now be published.

© 2021. Published by The Company of Biologists under the terms of the Creative Commons Attribution License (https://creativecommons.org/licenses/by/4.0/).
